# Combination of GC-MS, UHPLC-MS, and NMR Reveals Metabolic Differences Between the Oleo-Gum Resins of *Ferula tadshikorum* Pimenov and *F. foetida* (Bunge) Regel

**DOI:** 10.3390/plants15182781

**Published:** 2026-09-11

**Authors:** Nilufar Z. Mamadalieva, Michal Šoral, Olim K. Khojimatov, Davlat K. Akramov, Andrej Frolov, Ludger A. Wessjohann

**Affiliations:** 1 Institute of the Chemistry of Plant Substances, Uzbekistan Academy of Sciences, M. Ulugbek Str 77, Tashkent 100170, Uzbekistan; n.mamadaliyeva@afu.uz (N.Z.M.); a.davlat@inbox.ru (D.K.A.); 2 Department of Pharmacy and Chemistry, Faculty of Dentistry and Pharmacy, Alfraganus University, Yuqori Qoraqamish Str. 2a, Tashkent 100190, Uzbekistan; 3 Department of Bioorganic Chemistry, Leibniz Institute of Plant Biochemistry, Weinberg 3, 06120 Halle (Saale), Germany; afrolov@ipb-halle.de; 4 Analytical Department, Institute of Chemistry, Slovak Academy of Sciences, Dúbravská cesta 9, SK-845 38 Bratislava, Slovakia; michal.soral@savba.sk; 5 Institute of Botany, Uzbekistan Academy of Sciences, Durmon Yuli Str 32, Tashkent 100125, Uzbekistan; olimchik@mail.ru; 6 Laboratory of Analytical Biochemistry and Biotechnology, K.A. Timiryazev Institute of Plant Physiology of Russian Academy of Science, Botanicheskaya street 35, 127276 Moscow, Russia

**Keywords:** *Ferula tadshikorum*, *Ferula foetida*, HS-GC-MS, NMR, structure assignment, UHPLC-MS

## Abstract

**Background**: Central Asia is a key center of diversity for *Ferula* species, including *Ferula foetida* and *Ferula tadshikorum*, which are important but insufficiently characterized sources of oleo-gum resins. **Methods**: The oleo-gum resins of *F. tadshikorum* (OGRFT) and *F. foetida* (OGRFF) were analyzed using HS-GC-MS, UHPLC-MS, and NMR. **Results**: A total of 40 compounds were detected by HS-GC-MS, representing 94% and 97% of the volatiles in *F. foetida* and *F. tadshikorum*, respectively. The major volatile constituents of the resins were disulfides. RP-UHPLC-ESI-QqTOF-MS/MS tentatively identified 51 compounds, including sesquiterpene coumarins, sesquiterpenes, sulfides, phenolics and fatty acids in both OGRFT and OGRFF. NMR (1D and 2D) confirmed the identity of five major compounds and provided complete signal assignments for their potential quantification. UHPLC-MS revealed that OGRFF was rich in foetisulfide A, followed by daucane sesquiterpenes and esters of sesquiterpenes and fatty acids, while OGRFT was dominated by galbanic acid and 8-acetoxy-5-hydroxyumbelliprenin, as confirmed by both UHPLC-MS and NMR. Anthelmintic, antifungal, and cytotoxic assays demonstrated that the oleo-gum resins were non-toxic, at least in the in vitro test systems addressed. **Conclusions**: This study provides the first comprehensive metabolite profiling of oleo-gum resins from *F. foetida* and *F. tadshikorum*, revealing distinct chemical compositions and supporting the preferential use of *F. tadshikorum* in food applications. Advanced analytical approaches (HS-GC-MS, UHPLC-MS/MS, and NMR) identified key marker compounds, particularly sesquiterpene coumarins, enabling reliable differentiation, standardization and quality control.

## 1. Introduction

Central Asia, particularly its hilly regions, is the area where the family Apiaceae (alt. Umbelliferae) is best represented in the world, both in terms of biomass and species diversity. Within this family, the genus *Ferula* L., with more than 185 species, is the third largest one [1]. It has a wide distribution all over Central and South-West Asia (especially Uzbekistan, Iran, and Afghanistan), the Far-East, North India, and the Mediterranean, and some species are distributed in desert areas [2]. Altogether, 114 of the 185 *Ferula* species can be found in Central Asia. About 60 of them are described in the Uzbekistan Flora, where they occupy sandy deserts, hills, mountains, and foothills of the country with the highest representation in the Tashkent, Surkhandarya, Kashkadarya, Jizzakh, Navoi, and Bukhara regions and in the Republic of Karakalpakstan [3].

The majority of the *Ferula* plants have a pungent odor with some species used as a spice for centuries. The genus is characterized by the presence of oleo-gum resins, in particular those obtained from *Ferula assa-foetida* L., *F. alliacea* Boiss., *F. foetida* (Bunge) Regel, *F. narthex* Boiss. (asafoetida), *F. persica* Willd., *F. szowitsiana* DC. (sagapenum), *F. gummosa* Boiss. (galbanum), *F. moschata* (H.Reinsch) Koso-Pol. (sumbul), and *F. tingitana* L. and *F. communis* L. (African ammoniacum) [2]. In Iran, Pakistan, Afghanistan and India, gum and roots of *Ferula* are used as a seasoning in the food industry and as raw material for perfumes in the cosmetic industry [4]. Asafoetida is one of the most well-known flavoring spices widely used in the cuisines of South and South-West Asia for pickling, as a digestive aid, and as a condiment in meals [5].

*Ferula assa-foetida* is a *Ferula* species native to Iran and introduced into Bangladesh, Kazakhstan, Laos, Libya, Turkmenistan, and Uzbekistan (according to https://powo.science.kew.org). Although *F. assa-foetida* is universally believed to be the principal source of the oleo-gum resin in the global market, it should not be underestimated that several other *Ferula* species might act as the major gum sources in regional markets, and due to their misidentification they are often sold as *F. assa-foetida*. In fact, the production of oleo-gum resin from *F. assa-foetida* is confined to its native range, namely Iran, whereas other species can serve as the sources of “asafoetida” in other regions.

In Uzbekistan, clearly *F. foetida* (Bunge) Regel (subgenus *Scorodosma* Bunge) and *F. tadshikorum* Pimenov (subgenus *Narthex* Falc.) are the main sources of the oleo-gum resin (Figures S1–S4) and not *F. assa-foetida*. The commercial collection and export of *Ferula* gum from these Uzbek species started in 2002. However, the use of the resins so far is very limited in Uzbekistan, and they are mainly exported to Afghanistan, India, and China. Currently, the annual export of the oleo-gum resins from *F. foetida* and *F. tadshikorum* in Uzbekistan is approximately 100–400 tons.

The oleo-gum resin and dried powder of *F. foetida* have been used in Central Asian folk medicine as an anticonvulsant, vermifuge and neuroactive therapeutic agent. Avicenna used this plant to treat tumors, jaundice, and other liver diseases. Moreover, it was reported for the treatment of stomach, kidney, and spleen disorders. Finally, it was also used as a diuretic, and as a hemostatic agent against uterine bleeding [6]. *F. tadshikorum* has a long traditional use by local populations for the treatment of digestive disorders. In early spring, the young leaves are harvested, dried, and consumed on an empty stomach, often mixed with sour milk or suzma, to stimulate gastric secretion and improve digestive function. The plant resin is traditionally applied for the treatment of colds, persistent cough, chronic bronchitis, and for strengthening immunity. For this purpose, a small amount of resin is dissolved in warm water and taken orally two to three times daily after meals. The resin is also used as a remedy for toothache. In folk medicine, an alcoholic tincture prepared from the roots is used to relieve foot pain. Roots of medium-aged virginial plants are preferably collected, cleaned, cut into small pieces, and placed in a dark glass container filled with alcohol. The tincture is then stored in a dark place for approximately 10 days before use [7]. Since ancient times, the species also has been used as an analgesic agent to treat arthritis and joint pain. The communities of Central Asia use the resin of both *Ferula* species as a remedy to treat hysterics, neurasthenia and vegetative neurosis. They are also successfully employed in the therapy of multiple skin diseases and common colds, as an expectorant and anticonvulsant. Moreover, in the mixture with other drug substances, *Ferula* gums are employed in treatments of lung tuberculosis, exudative diathesis, lymphadenitis, and otitis. Some studies have shown an effect of *Ferula* preparations in the treatment of malignant tumors and syphilis. In this context, the leaves of *F. tadshikorum* are mixed with acidic milk [8].

Thus, the two *Ferula* species are a rich source for applications such as spices and in functional foods, nutraceuticals, and pharmaceuticals. Especially for the latter applications, to transfer hearsay health claims to scientifically proven effects, a better and complete analysis of all metabolites present in the different species and their organs is the crucial basis for any science-founded application, as well as for quality control and adulteration avoidance.

The significant advantages of NMR-, UHPLC-MS- and GC-MS-based metabolite profiling is the possibility to screen mixtures of plant secondary metabolites, like crude extracts or essential oils, for the presence of active molecules/biomarkers without preceding fractionation steps. Recent studies on the genus *Ferula* have demonstrated the effectiveness of untargeted and multi-platform metabolomics approaches (including GC-MS, UHPLC-MS/MS and NMR) for the characterization and differentiation of species-specific metabolites, often reporting hundreds of detectable compounds and chemotaxonomic markers across species and environmental conditions [9,10,11]. It is important to obtain a comparative understanding of the chemical components of various “asafoetida” sources, especially of “newer” entries to the world markets such as the oleo-gum resins of *F. foetida* (OGRFF) and *F. tadshikorum* (OGRFT) from Uzbekistan. Although both are traditionally used as sources of oleo-gum resins in Central Asia, their suitability for food-related applications may differ substantially due to variations in chemical composition. In particular, sulfur-containing volatile compounds are responsible for the characteristic pungent odor of asafoetida-like products and strongly influence sensory quality and consumer acceptability. Therefore, comparative metabolomic characterization of these species and their traded gums is important to capture their full potential in the food and related industries. It can be used for quality control, e.g., for chemotaxonomic purposes and to detect adulteration.

Accordingly, the aims of this research were the development of analytical methods for metabolic fingerprinting and profiling of the OGRFF and OGRFT and the establishment of their gas chromatography–mass spectrometry (GC-MS), reversed phase ultra-high-performance liquid chromatography (RP-UHPLC-QqTOF-MS) and nuclear magnetic resonance (NMR) spectroscopy profiles. In addition, oleo-gum resins were further evaluated for their potential as antifungal, anthelmintic and cytotoxic agents.

## 2. Results and Discussion

### 2.1. HS-GC-MS Analysis

Here, to the best of our knowledge, for the first time, we provide comprehensive characterization of the volatile compounds obtained from the OGRFF and OGRFT collected in Uzbekistan. Based on the results of the spectral similarity search, 25 and 32 compounds were identified in the resin obtained from *F. foetida* and *F. tadshikorum*, respectively. In these preparations, the identified compounds accounted for 94% and 97% of the total integrated peak area, respectively. The peak area percentages attributed to individual identified constituents and the corresponding retention indices are listed in Table 1. Gas chromatograms of the volatile compounds obtained from the oleo-gum resins of *F. foetida* and *F. tadshikorum* are shown in Figures S5 and S6.

The HS-GC-MS studies revealed that for both resins, sulfur-containing compounds form a major part of the volatile metabolome. In the composition of the OGRFF volatile fraction, sulfur-containing metabolites accounted for 76% of the total integrated peak area. The major constituents of OGRFF were bis(1-methylpropyl) disulfide (**33**, 54.4%), 2-(isopropyldisulfanyl)butane (**29**, 13.2%), (*E*)-*β*-ocimene (**25**, 9.8%), (*Z*)-*β*-ocimene (**24**, 2.9%), *α*-pinene (**12**, 2.9%) and isopropyl disulfide (**21**, 2.0%). In turn, the volatile fraction of OGRFT (overall 93% of the total integrated peak area) was dominated by the sulfur-containing compounds *S*-(*E*)-1-propenyl *S*’-1-methylpropyl disulfide (**32**, 42.0%), *S*-(*Z*)-1-propenyl *S*’-1-methylpropyl disulfide (**31**, 39.0%), *S*-propyl *S*’-1-methylpropyl disulfide (**30**, 5.34%) and *S*-(1-(methylthio)propyl) *S*’-(*Z*)-1-propenyl disulfide (**37**, 4.50%). In OGRFT, terpenoids were much less represented (3.30% of the total peak area), with *α*-pinene (**12**, 1.1%) being the most abundant terpene metabolite. As can be seen from Table 1, OGRFF and OGRFT demonstrate considerable qualitative and quantitative differences in their chemical profiles. For example, 2-(isopropyldisulfanyl)butane (**29**) and isopropyl disulfide (**21**) were not found in *F. tadshikorum* resin, while they were the abundant in *F. foetida* resin (13.2% and 2.0%, respectively).

*F. tadshikorum* is known to be the main source of the oleo-gum resin in Uzbekistan. A detailed chemical composition of the essential oil obtained from the underground parts of *F. tadshikorum* collected in the southern part of Tajikistan was recently reported by Sharopov et al. [12]. According to the authors, the major constituents of this oil were the sulfur-containing compounds *S*-(*Z*)-1-propenyl *S*’-1-methylpropyl disulfide (**31**, 37.3%), *S*-(*E*)-1-propenyl *S*’-1-methylpropyl disulfide (**32**, 29.9%), *S*-(1-(methylthio)propyl) *S*’-(*E*)-1-propenyl disulfide (**38**, 16.8%), and *S*-propyl *S*’-1-methylpropyl disulfide (**30**, 4.8%). Two of these disulfides, **31** and **32**, are the principal components of many *Ferula* species, especially in the resin of *F. assa-foetida*, as was repeatedly reported [13,14]. Our findings demonstrated that the patterns of volatile compounds in the OGRFT and the chemical composition of the essential oils extracted from underground parts of this species by Sharopov et al. [12] are quite similar. However, the relative abundance of the two biomarker compounds **31** and **32** was higher in the OGRFT of our collection from Uzbekistan.

Organosulfur compounds serve as chemotaxonomic markers for the characterization of *Ferula* species. Six sulfide derivatives, named foetisulfides A-D and foetithiophenes A and B, were isolated from the ethyl acetate-soluble fraction of *F. foetida* methanolic root extracts [15,16]. Khalilova et al. [17] also studied ethanolic extracts and the juice of aerial parts of *F. foetida* by GC-MS, showing that they contained 2,3,4,5-tetramethylthiophene and 2-ethylthiopyridine as principal constituents, while ethanolic extracts contained 1,3-dimethyltrisulfane, 1,8-cineol (eucalyptol), *S*-methylmethanethiosulfonate, 2,3,4,5-tetramethylthiophene, (+)-*trans*-chrysanthenylacetate, (+)-calarene, bulnesol, etc. Our findings obtained here with the *F. foetida* oleo-gum resin were different from the abovementioned reports dealing with alcoholic extracts, fractions and juice obtained from the roots and aerial parts of *F. foetida* [16,17].

### 2.2. RP-UHPLC-MS Analysis

The total ion chromatograms of OGRFF and OGRFT acquired in positive and negative ion modes by RP-UHPLC-QqTOF-MS/MS are shown in Figures S7 and S8. Based on a feature analysis, individual metabolites were annotated and their structures were preliminarily assigned by SWATH-MS/MS (Table 2 and Table 3). Annotation of metabolites relied on the experimental RP-UHPLC-QqTOF-MS data (accurate mass, isotopic distribution, and fragmentation pattern) [18,19] and their detailed consideration in the context of data from the literature on natural compounds observed in the *Ferula* genus earlier.

A total of 51 compounds were tentatively assigned for the oleo-gum resins. In positive ion mode, most of the analytes were observed as [M+H]^+^, [M+NH_4_]^+^, [M+Na]^+^, [2M+H]^+^, and [2M+NH_4_]^+^ adducts. On the other hand, in negative ion mode, formation of formate adducts ([M+HCOO]^−^) was universally observed. In addition, intense quasimolecular ions of dimers ([2M−H]^−^) were observed. The primary annotation relied on tR and exact *m*/*z* with further confirmation by interpretation of MS/MS spectra. Based on this strategy, altogether 28 sesquiterpene coumarins, three further sesquiterpenes and their derivatives, two phenolics, and one fatty acid were assigned in the OGRFT, while eight mono- and sesquiterpene coumarins, five sesquiterpenes and derivatives, two sulfides, three fatty acids and derivatives, and two phenolics (besides coumarins) were annotated in the OGRFF (Table 2). Chemical structures of the identified major constituents of the oleo-gum resins are shown in Figures S9 and S10.

*Sesquiterpene coumarins.* Sesquiterpene coumarins are meroterpenoids that are chimeras of a common coumarin group and a sesquiterpene moiety of diverse structures. The *Ferula* genus is known as the major source of sesquiterpene coumarins. The chemical constituents of this genus were intensively studied over the last 30 years. For example, one of the most comprehensive studies of Gliszczyńska and Brodelius [20] revealed around 130 different sesquiterpene coumarins. However, the sesquiterpene coumarins from the species *F. tadshikorum* and *F. foetida* were not addressed in such detail so far. Therefore, to the best of our knowledge for the first time, herein 28 sesquiterpene coumarins in the oleo-gum resins of *F. tadshikorum* are characterized by RP-UHPLC-ESI-MS/MS (Table 2).

LC-MS of OGRFT analyses in both positive and negative ion mode revealed 8-acetoxy-5-hydroxyumbelliprenin (**57**), galbanic acid (**58**), farnesiferol A (**62**), karatavicinol (**53**), assafoetidnol A (**48**), feselol (**65**), 10’*R*-acetoxy-11’-hydroxyumbelliprenin (**59**), and 5,8-dihydroxyumbelliprenin (**54**) as the major compounds. These could be tentatively assigned by tR-s and exact masses. Among them, 8-acetoxy-5-hydroxyumbelliprenin (**57**, C_26_H_32_O_6_, *m*/*z* 485.2181 [M+HCOO]^−^, *m*/*z* 458.2545 [M+NH_4_]^+^, *m*/*z* 441.2272 [M+H]^+^) and galbanic acid (**58**, C_24_H_30_O_5_, *m*/*z* 399.2188 [M+H]^+^, *m*/*z* 397.2020 [M−H]^−^) yielded the highest signal intensities in both ionization modes and are, therefore, proposed as the principal components of the OGRFT (Figures S11 and S12). Remarkably, in the negative ion mode, compound **58** yielded a signal of the base deprotonated ion [M−H]^−^ at *m*/*z* 397.2020, which was accompanied by an intense signal of the [M+HCOO]^−^ ion at *m*/*z* 443.2052. Tandem mass spectrometry (MS/MS) provided an additional level of metabolite annotation. Thus, for the compound **58**, the signal of the fragment ion at *m*/*z* 163.0392 [C_9_H_7_O_3_]^+^, formed from 7-hydroxycoumarin by the loss of the C_15_ sesquiterpene unit, could be clearly seen in the spectra. Furthermore, the signal at *m*/*z* 237.1857 corresponds to this moiety ([C_15_H_25_O_2_]^+^). Along with the exact mass of the precursor [M+H]^+^ ion (signal at *m*/*z* 399.2188), this fragmentation pattern confirms the assignment of the compound **58** as galbanic acid.

The second major compound of the *F. tadshikorum* extracts was assigned as 8-acetoxy-5-hydroxyumbelliprenin (**57**) (Figure S11). This annotation relied on the corresponding negative ion tandem mass spectrum of the formate adduct ion ([M+HCOO]^−^) signal at *m*/*z* 485.2181. It represents a fragment signal at *m*/*z* 161.0243, which corresponded to the 7-hydroxycoumarin moiety. In positive ion mode, this compound exhibited the major molecular ion signals as a [M+NH_4_]^+^ adduct at *m*/*z* 458.2554, and [M+H]^+^ at *m*/*z* 441.2272. Importantly, the absolute majority of the sesquiterpene coumarins, which could be identified here by RP-UHPLC-MS and MS/MS, were detected in the OGRFT for the first time (Table 2). There are some early reports on chemical constituents (tadzhiferin, tadzhikorin, deacetyltadzhikorin) from *F. tadshikorum* [21], which to the best of our knowledge could be misidentified. We also found that the OGRFF was featured with lower structural diversity of sesquiterpene coumarins and with lower relative contents of individual constituents, as compared with OGRFT. Indeed, only microlobin (**80**), 8-acetoxy-5-hydroxyumbelliprenin (**57**), galbanic acid (**58**), a microlobin derivative (**81**), and umbelliprenin (**74**) were found in the former species. Thus, our results are in good agreement with earlier reports, e.g., from Abd El-Razek et al. [22], who isolated sesquiterpene coumarin **57** from the methanol extract of the OGRFF.

*Sesquiterpenes and derivatives*. The *Ferula* genus is well documented as an excellent source of biologically active compounds such as daucane, humulane, himachalane, and guaiane sesquiterpenes and their derivatives. Previous chemical studies on *Ferula* species showed that the major natural products occurring in their extracts are daucane-type sesquiterpenes [23]. An RP-UHPLC-QqTOF-MS/MS investigation of the OGRFF and OGRFT showed high relative abundances of five and three sesquiterpene derivatives, respectively. Among the derivatives demonstrating higher abundance in OGRFF were daucane (carotane) sesquiterpenes and their esters, daucane sesquiterpene 1-acetate, 5-isovalerate of lapiferol (**83**, C_22_H_36_O_6_, *m*/*z* 441.2494 [M+HCOO]^−^) and 3-angelate of felikiol (**87**, C_20_H_32_O_4_, *m*/*z* 381.2283 [M+HCOO]^−^). On the other hand, the daucane kuhistanicaol A (**46**), as well as sesquiterpene esters vesceritenone (**50**) and 2,10-diacetyl-8-hydroxyferutriol-6-anisate (**51**), were found in the OGRFT.

*Sulfides.* It is well-known that sulfur-containing compounds are responsible for the characteristic onion-like odor of *Ferula* oleo-gum resins. Among these, polysulfides have been reported as major constituents of the oleo-gum resins of several *Ferula* species, particularly *F. assa-foetida* [15,24,25,26,27,28], and *sec*-butyl sulfides are widely recognized as chemotaxonomic markers of the genus. Based on the results of the RP-UHPLC-MS analysis, the major peak in the methanolic extract of OGRFF was assigned to compound **77**, which was represented by a [M+H]^+^ ion at *m*/*z* 225.0444 in positive ionization mode. It was assigned as a foetisulfide A isomer (C_8_H_16_OS_3_) (Figure S13). Neutral sulfur-containing compounds (e.g., thioethers, sulfides, and sulfur-containing sesquiterpenes) generally ionize more efficiently in positive electrospray ionization mode due to their higher proton affinity and tendency to form stable protonated or adduct ions [29]. In contrast, such compounds typically show weak response or may not be detected under negative ionization conditions; therefore, the peak at tR 9.8 min corresponding to foetisulfide A (**77**) was not observed in negative ionization mode under the experimental conditions used. In turn, compound **76** was assigned as another isomer of foetisulfide A (C_8_H_16_OS_3_, [M+H]^+^ at *m*/*z* 225.0436). One of these isomers (most likely compound **77**) was previously isolated from a methanolic extract of *F. foetida* collected from Uzbekistan [24].

*Phenolic compounds.* Phenolic acids, catechins, flavonoids, tannins, stilbenes, lignans, and anthocyanidins are widely found in plants. They play an important role in UV protection and disease prevention [30,31]. The phenolic compounds, which were annotated in the OGRFF and OGRFT, are summarized in Table 2 and Table 3, respectively. Two phenolic compounds, ferulic acid (**41**, C_10_H_10_O_4_, *m*/*z* 193.0501 [M−H]^−^) and coniferyl ferulate (**45**, C_20_H_20_O_6_, *m*/*z* 355.1187 [M−H]^−^), were tentatively identified in the OGRFT. In the present work, cinnamic acid (**75**, C_9_H_8_O_2_, [M+HCOO]^−^ at *m*/*z* 193.0506) and 2-epihelmanticine (**86**, C_26_H_34_O_10_, [M−H]^−^ at *m*/*z* 505.2079) were tentatively identified and for the first time reported for the OGRFF.

*Fatty acids and derivatives*. Oleo-gum resins are more or less homogeneous mixtures of gum, volatile oil, and resin. Surprisingly, the fatty acid composition of the OGRFF and OGRFT had not been reported so far. Therefore, here we addressed this question by RP-UHPLC-MS. By this approach, saturated tetradecanoic acid (syn. myristic acid) (**71**, C_14_H_28_O_2_, [M−H]^−^ at *m*/*z* 227.2017) could be annotated in the OGRFT (Table 2). Earlier, myristic acid was found to be the major fatty acid component in *F. communis* L. stems [32]. In this study, three fatty acids, namely pentadecenoic acid (**89**, C_15_H_28_O_2_, [M−H]^−^ at *m*/*z* 239.2017), palmitic acid (**90**, C_16_H_32_O_2_, [M−H]^−^ at *m*/*z* 255.2330) and octadecenoic acid (**91**, C_18_H_34_O_2_, [M−H]^−^ at *m*/*z* 281.2486), were found to be the major compounds in OGRFF (Table 3). In Youssef et al. [33], palmitic acid (39.03%) was reported as the major component in *F. samarcandica* Korovin. Similarly, the fatty acid composition of OGRFF observed in the study exhibited a comparable distribution pattern to that reported previously (Table 2 and Table 3).

Recent advances in untargeted metabolomics, particularly UHPLC-MS/MS, GC-MS, LC-QTOF-MS, and NMR-based approaches, have significantly improved the characterization of secondary metabolites in *Ferula* species. These techniques enable comprehensive profiling of volatile and non-volatile metabolites and facilitate discrimination among closely related species. Several studies published during 2023–2025 summarized recent phytochemical and metabolomic progress in *Ferula*. Chen et al. [34] reviewed the phytochemistry of *F. ferulaeoides* and emphasized the diversity of volatile oils, terpenoids, and coumarins identified using chromatographic and spectrometric techniques. Similarly, Wang et al. [35] summarized sesquiterpene diversity and biosynthetic pathways in *Ferula*, highlighting the importance of metabolomics and LC-MS technologies in the discovery of novel sesquiterpene derivatives.

A recent metabolomic investigation by Jiang et al. [9] applied untargeted UHPLC-MS/MS metabolomics to five *Ferula* species from Central Asia. The researchers identified 764 metabolites and screened 56 characteristic biomarkers. The study demonstrated that environmental factors, including geography and ecological conditions, significantly influence metabolite accumulation. Species-specific variations were particularly evident in terpenoids, phenolics, flavonoids, and organic acids, indicating that both genetic diversity and habitat conditions contribute to metabolic heterogeneity in *Ferula* species.

Another important study by Jiang et al. [36] investigated four species using HS-SPME/GC-MS and UHPLC-MS/MS-based untargeted metabolomics. The authors identified 166 volatile organic compounds in leaves and 1079 metabolites in roots. Sulfur-containing compounds, aldehydes, ketones, terpenoids, amino acids, and fatty acids were identified as the major discriminatory metabolites. The study also demonstrated species-specific metabolite accumulation patterns and suggested that root metabolites contribute directly to the characteristic aroma of aerial parts.

A recent study by Tian et al. [10] combined volatile metabolomics with transcriptomic analysis of *F. sinkiangensis* and *F. ferulaeoides*. A total of 561 volatile organic compounds were annotated, with volatile sulfur compounds predominating in *F. sinkiangensis* (45.98%) and terpenoids prevailing in *F. ferulaeoides* (44.01%). Using SPME-GC-MS, the authors characterized volatile compounds associated with odor formation and linked metabolite accumulation to differential expression of biosynthetic genes.

Untargeted metabolomic analysis of *F. violacea* Korovin identified 540 metabolites, including 419 compounds newly reported for the *Ferula* genus [37]. Terpenoids, alkaloids, and amino acid derivatives were the major metabolites, with roots enriched in terpenoids and seeds containing higher levels of alkaloids and amino acids. The study highlighted the rich phytochemical diversity and pharmacological potential of *F. violacea*.

**Table 2:** Compounds identified in the methanolic extracts obtained from oleo-gum resin of *F. tadshikorum* (OGRFT) by UHPLC-QqTOF-MS/MS *.

Compound	tR (min)	Tentative Annotation	Ion	Elemental Composition	*m*/*z*	Mass Accuracy (ppm)	Fragmentation Pattern	Compound Class	Other Source Organisms Reported in the Literature	References
**41**	6.1	Ferulic acid	[M−H]^−^	C_10_H_10_O_4_	193.0501	2.4	178.0263, 161.0240, 149.0605, 134.0371	phenolic	*F. assa-foetida* L.	[38]
**42**	9.8	Samarcandicin G	[M+H]^+^	C_26_H_34_O_7_	459.2377	0.4	423.2148, 441.2269, 399.2164, 381.2059, 363.1945, 237.1470, 219.1738, 201.1636, 163.0381	sesquiterpene coumarin	*F. samarkandica* Korovin	[39]
**43**	10.3	Samarcandin	[M+H]^+^	C_24_H_32_O_5_	401.2323	1.9	383.2218, 365.2099, 221.1905, 203.1799, 163.0387	sesquiterpene coumarin	*F. samarcandica*, *F. drudeana* Korovin, *F. tenuissima* Hub.-Mor. & Peșmen, *F. huber-morathii* Peșmen, *F. duranii* Sağıroğlu & H.Duman, *F. assa-foetida*	[40,41,42]
**44**	10.9	Samarcandicin G isomer	[M+HCOO]^−^	C_26_H_34_O_7_	503.2287	−4.1	497.1590, 451.7272, 445.1297, 238.8915, 163.0230	sesquiterpene coumarin		
**45**	11.0	Coniferyl ferulate	[M−H]^−^	C_20_H_20_O_6_	355.1187	−4.5	193.0500, 149.0601, 134.0365	phenolic		
**46**	11.2	Kuhistanicaol A	[M−H]^−^	C_25_H_34_O_8_	461.2181	−3.0	443.2052, 238.8902, 193.0498, 161.0232	daucane sesquiterpene	*F. kuhistanica* Korovin	[43]
**47**	11.3	Kamolonol	[M+HCOO]^−^	C_24_H_30_O_5_	443.2075	1.1	461.2162, 413.1922, 260.8725, 161.0232	sesquiterpene coumarin	*F. assa-foetida*, *F. narthex*, *F. pseudalliacea* Rech.f.	[44,45]
			[M+H]^+^		399.2166	2.0	416.2439 [M+NH_4_]^+^, 381.2071, 327.1597, 219.1743, 201.1632, 163.0387			
**48**	11.5	Assafoetidnol A	[M+HCOO]^−^	C_24_H_30_O_5_	443.2075	1.4	248.0774, 161.0231	sesquiterpene coumarin	*F. assa-foetida*, *F. sinkiangensis* K.M.Shen	[46,47]
			[M+H]^+^		399.2153	−1.2	421.1994 [M+Na]^+^, 397.2011, 381.2043, 363.1946, 307.1322, 219.1746, 215.0695, 201.1625, 163.0387			
**49**	12.0	Kamolonol acetate	[M+H]^+^	C_26_H_32_O_6_	441.2272	0.5	399.2167, 381.2054, 327.1588, 311.1621, 237.1839, 219.1742, 201.1632, 163.0385	sesquiterpene coumarin	*F. pseudalliacea*	[45]
**50**	12.1	Vesceritenone	[M−H]^−^	C_25_H_34_O_8_	461.2181	−4.5	176.0095, 161.0228	sesquiterpene derivative		
**51**	12.4	2,10-Diacetyl-8-hydroxyferutriol-6-anisate	[M−H]^−^	C_27_H_36_O_9_	503.2287	−2.7	497.1650, 238.8900, 161.0223	sesquiterpene derivative	*F. vesceritensis*	[48]
**52**	12.6	Feropolin	[M+HCOO]^−^	C_26_H_36_O_7_	505.2443	−3.4	238.8889, 161.0232	sesquiterpene coumarin	*F. polyantha* Korovin	[49]
			[M+H]^+^		461.2534	−0.4	443.2435, 441.2273, 401.2332, 383.2221, 275.1261, 259.0967, 215.0700, 163.0384			
**53**	12.7	Karatavicinol	[M+HCOO]^−^	C_24_H_32_O_5_	445.2232	3.5	399.2171, 176.0106, 161.0228	sesquiterpene coumarin	*F. assa-foetida*	[38]
			[M+H]^+^		401.2323	1.6	383.2220, 365.2113, 221.1897, 203.1799, 163.0390			
**54**	13.2	5,8-Dihydroxyumbelliprenin (asacoumarin A)	[M+HCOO]^−^	C_24_H_30_O_5_	443.2075	1.1	297.1814, 265.1472, 238.8905, 161.0225	sesquiterpene coumarin	*F. assa-foetida*, *F. foetida*, *F. narthex*, *F. seravschanica* Pimenov & J.V.Baranova	[22,50,51,52]
			[M+NH_4_]^+^		416.2431	1.3	421.1991 [M+Na]^+^, 381.2054, 363.1961, 307.1328, 259.0972, 219.1741, 201.1636, 163.0389			
**55**	13.3	Assafoetidnol B	[M+H]^+^	C_26_H_32_O_7_	457.2221	−1.7	479.2029 [M+Na]^+^, 455.2426, 397.2005, 397.1902, 361.1800, 263.2007, 259.0966, 235.1691, 215.0686, 203.1780, 193.0483, 163.0387	sesquiterpene coumarin	*F. assa-foetida*, *F. sinkiangensis* K.M.Shen	[46,47]
**56**	13.6	5’-Acetoxy-8’-hydroxyumbelliprenin	[M+HCOO]^−^	C_26_H_32_O_6_	485.2181	2.5	457.2265, 311.1665, 238.8904, 192.0047, 161.0231	sesquiterpene coumarin	*F. assa-foetida*	[44]
**57**	13.7	8-Acetoxy-5-hydroxyumbelliprenin	[M+HCOO]^−^	C_26_H_32_O_6_	485.2554	2.5	192.0053, 161.0243	sesquiterpene coumarin	*F. assa-foetida*, *F. foetida*, *F. narthex*, *F. seravschanica*	[44,50,51,53]
			[M+H]^+^		441.2272	−0.8	458.2545 [M+NH_4_]^+^, 381.2068, 363.2020, 307.1330, 215.0708, 201.1634, 163.0397			
**58**	13.8	Galbanic acid (asacoumarin B)	[M+H]^+^	C_24_H_30_O_5_	399.2188	1.8	381.2066, 237.1857, 219.1745, 189.0546, 181.1225, 163.0392	sesquiterpene coumarin	*F. assa-foetida*, *F. violacea* Korovin *(F. eugenii* Kamelin Boiss., *F. kokanica* Regel & Schmalh., *F. szowitsiana (F. microloba* Boiss.), *F. sinkiangensis*, *F. persica* Willd., *F. violacea*	[54,55,56,57]
			[M−H]^−^		397.2020	−1.4	161.0261			
**59**	14.2	10’*R*-Acetoxy-11’-hydroxyumbelliprenin	[M+HCOO]^−^	C_26_H_34_O_6_	487.2337	−3.2	397.1973, 238.8868, 192.0036, 161.0229	sesquiterpene coumarin	*F. assa-foetida*	[44]
			[M+H]^+^		443.2428	1.5	465.2246 [M+Na]^+^, 425.2329, 383.2208, 365.2119, 281.2118, 263.2011, 221.1903, 203.1796, 163.0390			
**60**	14.5	Farnesiferol isomer 1	[M+HCOO]^−^	C_24_H_30_O_4_	427.2126	−3.8	425.1042, 415.2133, 361.1419, 325.1795, 192.0042, 161.0226	sesquiterpene coumarin	*F. assa-foetida*, *F. szowitsiana*	[47,58,59,60]
**61**	14.6	Fekrynol	[M+H]^+^	C_24_H_32_O_4_	385.2373	0.4	365.2112, 203.1794, 189.0539, 163.0387	sesquiterpene coumarin	*F. lehmannii* Boiss., *F. krylovii* Korovin, *F. pseudalliacea* Rech.f., *F. sinkiangensis*	[47,61]
**62**	14.8	Farnesiferol isomer 2	[M+NH_4_]^+^	C_24_H_30_O_4_	400.2482	1.9	405.2042 [M+Na]^+^, 365.2117, 285.1490, 231.1014, 215.0701, 203.1801, 163.0387	sesquiterpene coumarin	*F. assa-foetida*, *F. persica*, *F. sinkiangensis*	[47,58,59,60]
**63**	15.3	Sinkiangenol E	[M+H]^+^	C_26_H_32_O_6_	441.2272	−0.4	463.2087 [M+Na]^+^, 458.2540 [M+NH_4_]^+^, 381.2060, 363.1960, 219.1745, 201.1640, 163.0386	sesquiterpene coumarin	*F. sinkiangensis*	[47]
**64**	15.4	Farnesiferol isomer 3	[M+H]^+^	C_24_H_30_O_4_	383.2217	1.1	381.2064, 365.2113, 215.0687, 203.1796, 193.0170, 163.0384	sesquiterpene coumarin	*F. assa-foetida*, *F. flabelliloba* Rech.f. & Aellen, *F. gummosa* Boiss., *F. persica*, *F. pseudalliacea*	[45,46,59,62,63,64]
**65**	16.0	Feselol	[M+H]^+^	C_24_H_30_O_4_	383.2217	1.1	405.2043 [M+Na]^+^, 365.2116, 221.1894, 203.1798, 189.0537, 163.0391	sesquiterpene coumarin	*F. drudeana* Korovin, *F. tenuissima*, *F. Huber-morathii*, *F. duranii*, *F. assa-foetida*, *F. gummosa*	[40,65]
**66**	16.2	Conferone	[M+H]^+^	C_24_H_28_O_4_	381.2060	1.0	365.2104, 323.1641, 273.1119, 257.0809, 219.1740, 215.0701, 203.1787, 163.0382	sesquiterpene coumarin	Regel & Schmalh., *F. iliensis* Krasn. ex Korovin, *F. incisoserrata* Pimenov & J.V.Baranova, *F. korshinskyi* Korovin, *F. litvinowiana* Koso-Pol., *F. narthex*, *F. persica*, *F. seravschanica*, *F. moschata (H.Reinsch) Koso-Pol. (F. sumbul* (Kauffm.) Hook. f.), *F. teterrima* Kar. & Kir.	[66,67,68,69,70,71]
**67**	16.8	Farnesiferone B	[M+H]^+^	C_24_H_28_O_4_	381.2060	1.7	363.1956, 219.1747, 201.1639, 163.0388	sesquiterpene coumarin	*F. persica*, *F. sinkiangensis*	[64,72]
**68**	16.9	Badrakemin acetate	[M+H]^+^	C_26_H_32_O_5_	425.2323	−2.5	447.2148 [M+Na]^+^, 387.1938, 365.2117, 203.1794, 163.0381	sesquiterpene coumarin	*F. assa-foetida*, *F. paniculata* Ledeb. (*F. feruloides* Korovin)	[59,73]
**69**	17.1	Badrakemin acetate isomer	[M+H]^+^	C_26_H_32_O_5_	425.2323	−3.6	447.2142 [M+Na]^+^, 427.2479, 387.1931, 365.2118, 285.1487, 265.2172, 215.0701, 203.1800, 163.0392	sesquiterpene coumarin		
**70**	17.2	Fekrynol acetate	[M+H]^+^	C_26_H_34_O_5_	427.2479	0.2	385.2360, 367.2256, 265.2170, 229.0863, 205.1945, 189.0550, 163.0391	sesquiterpene coumarin	*F. krylovii* Korovin, *F. pseudalliacea*, *F. sinkiangensis*	[45,47,61,72]
**71**	17.3	Tetradecanoic acid	[M-H]^−^	C_14_H_28_O_2_	227.2017	−4.6	238.8891, 220.8771, 199.8481, 158.9233, 116.9265, 78.9578	fatty acid		
**72**	17.5	Conferoside	[M+NH_4_]^+^	C_30_H_38_O_10_	576.2808	4.9	581.3790 [M+Na]^+^, 541.3865, 481.3655, 463.3576, 421.3465, 403.3350, 285.2223, 271.2385, 201.1628, 147.1169	sesquiterpene coumarin glycoside	*F. conocaula* Korovin	[74]
**73**	18.1	Seravschanin D	[M+H]^+^	C_24_H_28_O_3_	365.2111	1.9	387.1926 [M+Na]^+^, 215.0695, 203.1789, 163.0389	monocyclic-type sesquiterpene coumarin	*F. seravschanica*	[51]
**74**	18.8	Umbelliprenin	[M+H]^+^	C_24_H_30_O_3_	367.2268	2.0	205.1953, 163.0391, 149.1322	sesquiterpene coumarin	*F. assa-foetida*, *F. Jaeschkeana*, *F. sinkiangensis*	[59,75,76]

* Compounds tentatively annotated based on QqTOF-MS data and data from the literature of natural compounds observed in the *Ferula* genus.

**Table 3:** Compounds identified in the methanolic extracts obtained from oleo-gum resin of *F. foetida* (OGRFF) by UHPLC-QqTOF-MS/MS *.

Comp	tR (min)	Tentative Annotation	Ion	Elemental Composition	*m*/*z*	Mass Accuracy (ppm)	Fragmentation Pattern	Compound Class	Other Source Organisms Reported in the Literature	References
**75**	6.1	Cinnamic acid	[M+HCOO]^−^	C_9_H_8_O_2_	193.0506	0.1		phenolics		
**76**	9.4	Foetisulfide A isomer	[M+H]^+^	C_8_H_16_OS_3_	225.0436	4.9	217.1075, 149.0214, 134.9921, 104.9817, 87.0258	sulfide		
**77**	9.8	Foetisulfide A	[M+H]^+^	C_8_H_16_OS_3_	225.0444	−4.4	217.1047, 168.9796, 161.0448, 134.9922, 104.9825, 87.0261	sulfide	*F. foetida*	[16]
**78**	12.9	Auraptene	[M−H]^−^	C_19_H_22_O_3_	297.1496	2.4	260.8730, 238.8914, 183.0122, 161.0445	monoterpene coumarin	*F. gummosa*, *F. szowitsiana* (*F. microloba* Boiss), *F. sinkiangensis*, *F. penninervis* Regel & Schmalh.	[57,77]
**79**	13.0	Ferulenol	[M−H]^−^	C_24_H_30_O_3_	365.2117	2.9	297.1525, 282.8554, 265.1484, 238.8915, 180.8904, 161.0439, 116.9286	sesquiterpene coumarin	*F. communis* L.	[78]
**80**	13.7	Microlobin	[M+HCOO]^−^	C_26_H_32_O_6_	485.2175	−1.6	311.1683, 260.8725, 238.8905, 201.9031, 146.9670, 116.9283	sesquiterpene coumarin	*F. microloba*, *F. assa-foetida*, *F. szowitsiana*	[57,79]
**57**	13.7	8-Acetoxy-5-hydroxyumbelliprenin	[M+H]^+^	C_26_H_32_O_6_	441.2272	−2.0	463.2085 [M+Na]^+^, 458.2539 [M+NH4]+, 363.1962, 201.1629, 163.0387	sesquiterpene coumarin	*F. foetida*, *F. assa-foetida*	[22,44]
**58**	13.9	Galbanic acid (asacoumarin B)	[M+H]^+^	C_24_H_30_O_5_	399.2188	1.8	421.1979 [M+Na]^+^, 416.2427 [M+NH_4_]^+^, 371.3154, 327.0078, 301.1408, 163.0392	sesquiterpene coumarin	*F. assa-foetida*, *F. sinkiangensis*, *F. szowitsiana*	[47,80]
**81**	14.0	Microlobin derivative	[M+HCOO]^−^	C_27_H_32_O_8_	529.2074	−0.8	279.2322, 249.0607, 238.8910, 113.0239	sesquiterpene coumarin		
**82**	14.4	Lapiferin derivative	[M+HCOO]^−^	C_20_H_32_O_5_	397.2232	2.6	325.1840, 238.8912, 158.9247, 116.9282, 96.9599	daucane sesquiterpene	*F. soongarica* Pall. ex Willd.	[81]
**83**	14.8	1-Acetyl lapiferol 5-isovalerate	[M+HCOO]^−^	C_22_H_36_O_6_	441.2494	2.7	325.1840, 116.9284, 96.9592	daucane sesquiterpene	*F. communis subsp. linkii* (Webb) Reduron & Dobignard *(F. linkii* Webb et Berthel)	[82]
**84**	15.1	Scopolin	[M−H]^−^	C_16_H_18_O_9_	353.0878	−0.3	340.2025, 282.8547, 263.0375, 258.8490, 219.0475, 204.0246, 116.9282	coumarin glycoside	*F. heuffelii* Griseb. ex Heuff., *F. elaeochytris* Korovin, *F. syreitschikowii* Koso-Pol.	[83]
**85**	15.2	Fervanol *p*-hydroxybenzoate	[M−H]^−^	C_22_H_28_O_3_	339.1966	4.6	325.1838, 320.8055, 183.0117, 116.9282	sesquiterpene ester	*F. lycia* Boiss., *F. haussknechtii* H.Wolff ex Rech.f.	[84,85]
**86**	15.5	2-Epihelmanticine	[M−H]^−^	C_26_H_34_O_10_	505.2079	4.3	497.1658, 482.7047, 444.7562, 382.7766, 339.2058, 337.2002, 238.8909, 216.8520, 116.9287	phenolic	*F. rigidula* Fisch. ex DC., *F. szowitsiana*	[77,86]
**87**	15.9	Felikiol 3-angelate	[M+HCOO]^−^	C_20_H_32_O_4_	381.2283	4.3	369.2463, 325.1834, 238.8912, 116.9285, 96.9607	daucane sesquiterpene	*F. hezarlalehzarica* Ajani, *F. communis subsp. linkii*	[82,87]
**88**	16.1	1-Acetate ferulinkiol 6-isovalerate	[M+HCOO]^−^	C_22_H_36_O_5_	425.2545	4.9	282.8572, 201.0946, 116.9273, 96.9600	daucane sesquiterpene	*F. communis subsp. linkii*	[88]
**89**	17.4	Pentadecenoic acid	[M−H]^−^	C_15_H_28_O_2_	239.2017	4.8	227.2028, 116.9280	fatty acid		
**74**	18.8	Umbelliprenin	[M+H]^+^	C_24_H_30_O_3_	367.2268	4.7	205.1935, 163.0389, 149.1326	sesquiterpene coumarin	*F. seravschanica*, *F. sinkiangensis*, *F. szowitsiana*, *F. persica*	[51,89]
**90**	19.2	Palmitic acid	[M−H]^−^	C_16_H_32_O_2_	255.2330	1.4	158.9243, 78.9583	fatty acid		
**91**	19.3	Octadecenoic acid	[M−H]^−^	C_18_H_34_O_2_	281.2486	0.3		fatty acid	*F. assa-foetida*	[44]

* Compounds tentatively annotated based on QqTOF-MS data and data from the literature of natural compounds observed in the *Ferula* genus.

Overall, recent studies have successfully integrated untargeted metabolomics, volatile profiling, transcriptomics, and chemometric analyses to characterize species diversity, ecological adaptation, aroma formation, and pharmacologically active metabolites in *Ferula*. Despite these advances, several challenges remain, including incomplete metabolite databases, difficulties in the structural annotation of novel terpenoids, and limited integration of metabolomics with genomics and proteomics.

### 2.3. NMR Analysis

Nuclear magnetic resonance spectroscopy has previously been employed in the identification and quantification process of secondary metabolites of several *Ferula* species [11,13,59]. In the present case, the 1D and 2D NMR spectra of OGRFF were dominated by two sets of signals (in a 5:2 molar ratio), with both of them containing signals characteristic of a *S*-(1-methylpropyl) (=*S*-*sec*-butyl) group. The major compound additionally contained a 1,3-disubstituted (*E*)-prop-1-enyl group, whereas the minor compound contained a 1,3-disubstited (*Z*)-prop-1-enyl group, bound to the second disulfide sulfur in each case. From the observed HMBC signals and from the ^1^H and ^13^C chemical shift values, it was also suggested that the latter two moieties were 1-substituted by an *S*-methyl group. Unbiased by the other experimental data and by the structures of isolated compounds described in the literature, a disulfide linkage has been suggested between the *sec*-butyl groups and position 3 of the 1-(methylsulfanyl)-prop-1-enyl groups. The resulting structures of the two compounds, 2-{[(2*E*)-3-(methylthio)-2-propen-1-yl]disulfanyl}butane (**92**) and 2-{[(2*Z*)-3-(methylthio)-2-propen-1-yl]disulfanyl}butane (**93**), resemble foetisulfide A (77)—the main sulfide observed within OGRFF by UHPLC-MS. The isolation of compounds **92** and **93** as a ca. 7:3 mixture from the resin *Asa foetida* was reported by Naimie et al. [90], and based on this finding, compound **92** was later synthesized from commercial (*E*)-1-(methylthio)-3-chloropropene [91]. The possibility of the presence of foetisulfide A (**77**) and its isomer **76** in the NMR sample instead of compounds **92** and **93** was considered. The hypothesis was discarded because of the *S*-Me group’s ^13^C nucleus’ high shielding (*δ_C_* = 13.7 ppm for **92** and *δ_C_* = 16.2 ppm for **93**). However, the presence of the *Z*-configuration in compound **93** provides a possibility that compound **76** could be the *Z*-isomer of foetisulfide A. No minor compounds were identified in the OGRFF NMR sample due to low concentrations and structural similarities (signal overlap).

Major differences in the ^1^H NMR spectra of OGRFF and OGRFT were readily observable—the spectrum of the OGRFT sample contained numerous multiplets throughout the whole aliphatic, olefinic and aromatic chemical shift regions, indicating a higher degree of diversity in the structures of its constituents (Figure S14). Furthermore, the presence of 8-acetoxy-5-hydroxyumbelliprenin (**57**), galbanic acid (**58**) and coniferyl ferulate (**45**) was proven with complete ^1^H and ^13^C signal assignments. The ^1^H and 2D NMR spectra contained the signals of six 1-substituted 1-(methylthio)-propyl groups with high relative integral intensity. Unfortunately, due to the lack of further homonuclear and heteronuclear correlations, it was not possible to identify these particular compounds. It was speculated that these signals could belong to a set of 1-(methylthio)propyl-1-propenyl disulfide stereoisomers or derivatives, two of which, *S*-(1-(methylthio)propyl), *S*’-(*E*)-1-propenyl disulfide (**38**) and *S*-(1-(methylthio)propyl), *S*’-(*Z*)-1-propenyl disulfide (**37**), were previously described by Yatham et al. [13]. Although a direct comparison of ^1^H and ^13^C chemical shifts is not possible due to the use of different deuterated solvents, the observed spectral patterns remain very similar. Khajeh et al. [92] described the presence of bis(1-methylthio)propyl disulfide (**36**) in the essential oil of *Ferula assa-foetida*—a compound that could easily correspond to one set of signals, especially as it was also observed by the HS-GC-MS method, but the unambiguous proof of its presence in the NMR sample was not possible due to the lack of reference data.

The absolute quantification of the compounds contained in OGRFF and OGRFT was not attempted, because this would have required a qualified sampling and extraction scheme, something we could not assure with the material obtained this time. However, a relative quantification of the extract “as obtained” is possible and is shown in Table S1. Absolute quantification can be easily accomplished using a proper line-fitting algorithm for the ^1^H NMR spectra, provided that either collection, resin generation and extraction are standardized, ensuring complete extraction into the NMR solvent, or—if complete extraction cannot be achieved—that a correction factor gained from spiked recovery experiments is applied. A list of peaks suitable for the quantification of selected compounds is provided in Table S1. The full signal assignment with signal multiplicities can be found in Tables S2–S6. It should be noted that the list of signals in Table S1 is not universally applicable as overlaps might occur due to the presence of secondary metabolites with similar NMR spectral patterns and due to lower operating frequencies of the NMR spectrometer. It is recommended to obtain a HSQC spectrum beforehand to identify any overlaps in the ^1^H NMR spectrum at a chosen frequency.

### 2.4. Biological Evaluations

#### 2.4.1. Antifungal Activity

Previous studies have shown that the essential oil isolated from the rhizome and roots of *F. gummosa* demonstrated pronounced antifungal activity against several human, animal and plant pathogenic fungi [93]. The studies also showed that the antifungal properties of different extracts of *F. hermonis* Boiss. were associated with high contents of the daucane aryl esters jaeschkeanadiol *p*-hydroxybenzoate (ferutinin) and jaeschkeanadiol benzoate (teferidin) [94,95]. Moreover, it is known that the extent of biological activities of *Ferula* extracts might depend on the geographic location and its ecological conditions [1].

The antifungal activity of the OGRFF and OGRFT were evaluated against the phytopathogens *Zymoseptoria tritici*, *Botrytis cinerea*, and *Phytophthora infestans* at concentrations of 42 and 125 μg/mL. The extracts appeared to be inactive against the oomycete *P. infestans* and were only very slightly active against fungi where OGRFF inhibited *Z. tritici* by 12% at 42 μg/mL and 33% at 125 μg/mL. OGRFT was even less active against *B. cinerea* and *S. triciti* (21.5% at 125 μg/mL and 12.6% at 125 μg/mL, respectively). Our findings revealed that the OGRFF and OGRFT had negligible or, in the base-case scenario, very weak antifungal activities against the plant pathogens tested. Our results are in agreement with those reported in previous studies, which indicated that oleo-gum resin of *F. assa-foetida* with higher sulfur content had only weak antifungal activities [96,97]. The low fungitoxic activity of the OGRFF and OGRFT might be due to the absence of ferutinin and teferidin in these samples [94].

#### 2.4.2. Anthelmintic Activity

*F. assa-foetida* extracts are traditionally used as an anthelmintic in Iran, China and Nepal. The anthelmintic properties of this species are well-characterized to date [4]. Recently, Tavassoli et al. [98] showed that on the first day of exposure, the aqueous alcoholic extract of *F. assa-foetida* killed over 91% of *Strongylus* spp. larvae when administered at a (rather high) concentration of 10 mg/mL. In the adult motility assay, 100% mortality of *Haemonchus contortus* was observed when crude aqueous methanolic extracts and ethyl acetate fractions of *F. assa-foetida* were applied at 12.5–50 mg mL^−1^ [99]. Aqueous extracts from the *F. foetida* resin were investigated by Gundamaraju [100] for their anthelmintic activity against *Pheretima posthuma*. The extract exhibited significant anthelmintic activity at a very high concentration of 100 mg/mL. This preparation still appeared to be more efficient than the drug piperazine citrate.

The OGRFF and OGRFT had not yet been tested against *Caenorhabditis elegans*, a non-pathogenic model test organism which can be used for the initial high-throughput screening of anthelmintic compounds. When tested against this nematode, OGRFF and OGRFT extracts did not show significant lethality even at a higher concentration (500 µg/mL), which is consistent with previous reports, indicating that *F. foetida* resin exhibits concentration-dependent anthelmintic activity only at extremely high doses (25–100 mg/mL) [100]. Although neither OGRFF nor OGRFT exhibited significant anthelmintic activity against *C. elegans* at the tested concentrations, these findings contribute to the biological characterization of *F. tadshikorum* oleo-gum resin. Considering its chemically well-defined composition and the potential for better standardization compared with other *Ferula* species, *F. tadshikorum* may represent a promising source of oleo-gum resin for future food and nutraceutical applications.

#### 2.4.3. Cytotoxic Activity

Several reports on cytotoxic activities of oleo-gum resin of *F. assa-foetida* are published so far, albeit also with quite high concentrations applied [101]. *Ferula* oil obtained from oleo-gum resin of *F. assa-foetida* and enriched with organosulfides displayed significant cell growth inhibition in in vitro tests with cultured SKOV3 and A549 cancer cells in a dose-dependent manner [13]. *F. assa-foetida* oleo-gum resin essential oil had the most pronounced cytotoxic effects against HeLa cell lines with an IC_50_ of 152 ± 3 µg/mL [2]. Recently, the cytotoxicity of the essential oil from the underground parts of *F. tadshikorum* was tested against CCRF-CEM and CEM/ADR5000 cancer cell lines: IC_50_ values were 21.6 µg/mL for CCRF-CEM and 142.5 µg/mL for CEM/ADR5000 cell lines [12]. Based on these data from the literature, it was decided to study the cytotoxic activity of the OGRFF and OGRFT against our standard cell lines PC-3 (prostate cancer) and HT-29 (colon adenocarcinoma). The results of the cytotoxic assays showed that the OGRFF and OGRFT did not show any activity against PC-3 and HT-29 cells at concentrations of 0.05 and 50 µg/mL in MTT (3-(4,5-dimethylthiazol-2-yl)-2,5-diphenyltetrazolium bromide) and CV (crystal violet) assays. Only OGRFF very moderately inhibited 14.6% of PC-3 cell growth at the highest concentration of 50 µg/mL. These findings indicate that, under the present experimental conditions and in the selected PC-3 and HT-29 cell models, OGRFF and OGRFT do not exhibit relevant cytotoxic activity.However, this does not exclude the possibility that different extracts, including those with anticancer properties described for some Ferula leaf preparations, concentrated applications of selected isolated compounds, or studies involving other cancer cell lines, may display different sensitivities. Our results are consistent with the previously reported low cytotoxicity of individual constituents isolated from *F. foetida* roots against certain cancer cell lines [15]. From the perspective of potential food and skin-care applications, the lack of cytotoxicity observed in the tested cell models may be considered a favorable characteristic.

## 3. Materials and Methods

### 3.1. Plant Materials

Oleo-gum resins of *F. tadshikorum* were collected from the mid-mountain region of the Dekhkanabad district of the Kashkadarya region, Uzbekistan (38.333189, 66.613782), in late June 2021. The *F. foetida* resins were collected from the flat desert area of the Kanimekh district of the Navoiy region, Uzbekistan (40.254421, 65.007704), in the middle of June. The habitats and collection sites do not overlap. The roots of the plants were scraped to collect their oleo-gum resins. All collected parts were frozen directly after harvesting and stored at −4 °C. Prof. Olim Khojimatov from the F.N. Rusanov Tashkent Botanical Garden of the Academy of Sciences of Uzbekistan identified the plants taxonomically (voucher specimens’ number DMP004521 and DMP01721). They are stored at the Institute of Botany, Academy of Sciences of Uzbekistan. Before proceeding with experiments, the samples were frozen.

### 3.2. Headspace Gas Chromatography–Mass Spectrometry (HS-GC-MS)

The HS-GC-MS method was adopted from Farhadi et al. [59] with changes. In detail, samples (*n* = 3, 2 g of each resin) in a 20 mL headspace vial were incubated at 100 °C for 30 min with constant shaking in a headspace assembly of the CTC GC PAL Injector (Shimadzu Deutschland GmbH, Duisburg, Germany). A total of 500 µL of each sample was injected on a ZB-5MS capillary column (30 m × 0.25 mm ID, 0.25 μm film thickness, Phenomenex, Aschaffenburg, Germany) and analyzed by gas chromatography–electron ionization–quadrupole mass spectrometry (GC-EI-Q-MS) using a GC2010 gas chromatograph coupled online to a quadrupole mass selective detector Shimadzu GC-MS QP2010 (Shimadzu, Kyoto, Japan). The temperature program started at 50 °C and was kept constant for (5 min at constant conditions), followed by two sequential ramps—3 °C/min ramp to 140 °C and 10 °C/min ramp to 240 °C. Afterwards, the temperature was kept constant at 240 °C for 10 min. MS detection was performed in the *m*/*z* range of 40–500. The analysis was performed under the control of GC-MS Real Time Analysis software (Shimadzu Deutschland GmbH, Duisburg, Germany). Standard alkanes (C_7_–C_40_, Sigma-Aldrich Darmstadt, Germany) were used to calculate the Kovats index (KI). Chromatograms were acquired using enhanced ChemStation software (version B.04.02, Agilent Technologies, Waldbronn, Germany). Volatile compounds were identified by spectral similarity search (with consideration of KI) against the Wiley Registry of mass spectral data and NIST library.

### 3.3. Sample Preparation and RP-UHPLC-QqTOF-MS Measurements

For preparation of the extracts, 4 mg of resin was macerated with 2 mL of LC-grade methanol using an ultrasonic bath for 10 min at room temperature, and afterwards it was centrifuged at 14,000× *g* for 10 min to remove debris. After centrifugation, the supernatant was transferred to vials (1 mL). As universally accepted for metabolomics studies [102], the resulting extracts were directly taken in the QqTOF-MS analysis, without determination of the dry residue contents. For the analysis, 5 µL of the OGRFF and OGRFT methanolic extracts was injected into an Acquity UPLC I class (Waters GmbH, Eschborn, Germany) and separated on a Nucleoshell RP18 column (150 mm × 2 mm × 2.7 µm; Macherey & Nagel, Düren, Germany) at 40 °C. Eluents A and B were aqueous 0.3 mmol/L ammonium formate and acetonitrile, respectively. The elution was accomplished at 5% eluent B for 2 min isocratically with a subsequent gradient to 95% eluent B in 17 min. After a 2 min long column washout at 95% eluent B and rapid eluent switch (to 5% eluent B in 0.1 min), the column was re-equilibrated at 5% eluent B during 5 min. The column flow rate was set to 0.4 mL/min and the autosampler temperature was 5 °C. The column effluent was transferred on line into an AB Sciex TripleTOF 6600 quadrupole time-of-flight (QqTOF) mass spectrometer equipped with a DuoSpray ion source (AB Sciex GmbH, Darmstadt, Germany), operated alternatively in positive and negative ion mode using a sequential window acquisition of all theoretical fragment-ion spectra (SWATH) algorithm. All analyses were performed at the high-sensitivity mode for both TOF-MS (MS^1^) and product ion (MS^2^) scans. The mass calibration was automatically performed every 10 injections using an APCI calibrant solution via a calibration delivery system (CDS) operated at 0.5 mL/min. The source operation parameters were set as follows: curtain gas, 55 psig; nebulizer (ion source gas 1), 60 psig; drying gas (ion source gas 2), 70 psig; drying gas temperature, 450 °C; ion spray voltage, 5.5/−4.5 kV; and declustering potential, 35 V. The TOF-MS scans were acquired in the mass range of 65–1250 *m*/*z* with the accumulation time of 100 ms. The MS^2^ scans were acquired with the accumulation time of 20 ms, a collision potential of 45/−45 V, a collision energy spread of 35 V, and a Q1 window of 25 *m*/*z*.

### 3.4. Sample Preparation for NMR Measurements and Processing

A total of 50 mg of the OGRFF and OGRFT was extracted with 1 mL DMSO-*d*_6_ (Deutero, Germany) containing 0.93 mmol/L hexamethyl disiloxane (HMDS) as standard, using an ultrasonic bath for 10 min followed by centrifugation for 15 min. Supernatants were placed in NMR tubes and were left to stand overnight at 24 °C. ^1^H NMR spectra of the DMSO-*d*_6_ extracts of the OGRFF and OGRFT were measured at 27 °C in a Varian VNMRS 600 NMR spectrometer (600 MHz) using 160 scans, a 0.274 s relaxation delay, a 2.726 s acquisition time, and a 30° pulse angle. ^1^H NMR spectra have been chemical shift-calibrated and phase- and baseline-corrected. Additionally, 2D NMR spectra were obtained from the DMSO-*d*_6_ extracts of the resins. All NMR raw data, acquisition parameters and processing parameters are available on request from the corresponding author. The ^1^H NMR spectra were integrated by the line-fitting algorithm included in the MNova 12 software package (Mestrelab Research, S.L., Santiago de Compostela, Spain).

### 3.5. Biological Assays

The antifungal, anthelmintic and cytotoxic activities of the OGRFF and OGRFT were evaluated by procedures described in our previous studies [19].

## 4. Conclusions

The oleo-gum resins of *Ferula foetida* and *Ferula tadshikorum* represent important traditional food additives and medicinal resources widely used in Central Asia and beyond. They are increasingly used instead of, or as substitutes for, or for additions to the oleo-gum of *F. assa-foetida*. In this study, a comprehensive metabolite profiling of these two species collected in Uzbekistan was performed using complementary HS-GC-MS, RP-UHPLC-QqTOF-MS/MS, and NMR spectroscopic approaches, providing the first integrated comparative characterization of the volatile and non-volatile metabolomes of their oleo-gum resins and basic bioactivity screens.

The obtained results clearly demonstrated pronounced qualitative and quantitative differences between the two species. *F. foetida* was characterized by a higher abundance of sulfur-containing volatile compounds, which are responsible for its typical pungent odor, whereas *F. tadshikorum* was enriched in sesquiterpene coumarins, particularly galbanic acid and 8-acetoxy-5-hydroxyumbelliprenin, which can be considered as key chemical markers of this species. These findings highlight distinct chemotaxonomic profiles and support the use of these metabolites for quality control and authentication of oleo-gum resin products.

Despite the marked chemical differences, both oleo-gum resins exhibited negligible or very weak in vitro antifungal, anthelmintic, and cytotoxic activities under the experimental conditions employed in this study. Specifically, no relevant cytotoxic effects were observed against the PC-3 and HT-29 cell lines within the tested concentration range. These findings indicate that neither resin exhibited pronounced acute cytotoxic or antimicrobial activity in the applied in vitro models. However, the biological activities of *Ferula* oleo-gum resins may depend on their chemical composition, the tested concentration, and the biological model employed. Therefore, the contribution of individual compound classes, particularly sulfur-containing metabolites and sesquiterpene derivatives, as well as potential selective activities toward other cell types, microorganisms, or mammals with their potential for metabolic modification of these constituents, warrants further investigation. The observed low cytotoxicity under the tested conditions, however, may be considered a favorable characteristic with respect to the potential use of these oleo-gum resins in food and skin-care applications.

The integration of GC-MS, LC-MS/MS, and NMR data enabled reliable identification of major constituents and confirmed the applicability of these techniques for rapid metabolomic fingerprinting of resins. In particular, sesquiterpene coumarins were identified as promising chemical markers for OGRFT, while sulfur-containing compounds dominated the metabolome of OGRFF. NMR is principally able to deliver at least relative semi-quantitative indications for the major compounds, but for exact quantification extraction, recovery and measurement parameters will have to be optimized.

Overall, the results will allow the production of chemically better defined and standardized oleo-gum resins for food and nutraceutical applications. *F. tadshikorum* resin is easily identifiable by its higher content of structurally valuable sesquiterpene coumarins and a lower proportion of highly odor-active sulfur compounds. In contrast, *F. foetida* remains characterized by a sulfur-rich volatile profile responsible for its strong sensory properties, which may be appealing for special food applications. Both species, fortunately, showed no or very low cell toxicity in vitro, supporting their general safety within the models tested and highlighting their potential for further investigation in functional and industrial applications. However, such an interpretation should be made with caution, since comprehensive safety assessment requires additional toxicological, pharmacological, and long-term in vivo studies.

## Figures and Tables

**Table 1:** Relative abundances of the volatile compounds identified in the oleo-gum resins from *F. foetida* and *F. tadshikorum*.

Peak	Elemental Composition	RI ^a^	RI ^b^	Compound	Peak Area, %	
					OGRFF	OGRFT
**1**	C_2_H_6_S_2_	768	771	Methyl disulfide	1.08	0.01
**2**	C_6_H_10_O	744	780	5-Hexen-3-one	0.00	0.01
**3**	C_7_H_8_	786	783	1,3,5-Cycloheptatriene	0.00	0.18
**4**	C_9_H_20_	823	818	2,4-Dimethylheptane	0.02	0.06
**5**	C_6_H_12_S	859	826	Allyl *n*-propyl sulfide	0.02	0.00
**6**	C_6_H_8_S	890	894	3,4-Dimethylthiophene	0.00	0.04
**7**	C_6_H_10_S	904	914	1-[1-Propenylsulfanyl]-1-propene	0.00	0.06
**8**	C_4_H_8_S_2_	923	919	Methyl *cis*-propenyl disulfide	0.00	0.01
**9**	C_6_H_10_S	907	921	Di(1-propenyl) sulfide	0.00	0.01
**10**	C_7_H_14_S	877	924	Allyl 1-methylpropyl sulfide	0.48	0.12
**11**	C_4_H_8_S_2_	928	928	Methyl 1-propenyl disulfide	0.00	0.12
**12**	C_10_H_16_	931	930	*α*-Pinene	2.88	1.08
**13**	C_4_H_8_S_2_	934	937	Methyl 2-propenyl disulfide	0.00	0.09
**14**	C_10_H_16_	943	945	Camphene	0.00	0.90
**15**	C_10_H_16_	970	969	Sabinene	0.00	0.48
**16**	C_10_H_16_	978	973	*β*-Pinene	0.60	0.12
**17**	C_10_H_16_	991	990	*β*-Myrcene	0.00	0.12
**18**	C_5_H_12_S_2_	995	992	Methyl -1-methylpropyl disulfide	0.36	0.02
**19**	C_10_H_16_	1007	1005	3-Carene		0.12
**20**	C_7_H_10_S	1002	1010	2,3,4-Trimethylthiophene	0.04	0.06
**21**	C_6_H_14_S_2_	1014	1017	Isopropyl disulfide	2.04	
**22**	C_10_H_14_	1025	1025	*o*-Cymene	0.36	0.00
**23**	C_10_H_16_	1029	1030	Limonene	0.06	0.00
**24**	C_10_H_16_	1036	1034	(*Z*)-*β*-Ocimene	2.88	0.18
**25**	C_10_H_16_	1047	1050	(*E*)-*β*-Ocimene	9.84	0.24
**26**	C_10_H_16_	1050	1061	*γ*-Terpinene	1.08	0.01
**27**	C_10_H_20_O_2_	1094	1109	3-Methylbutyl 2-methylbutanoate	0.07	0.00
**28**	C_10_H_20_O	1093	1110	*n*-Amyl isovalerate	0.10	0.00
**29**	C_7_H_16_S_2_	1119	1123	2-(Isopropyldisulfanyl)butane	13.2	0.00
**30**	C_7_H_16_S_2_	1172	1173	*S*-propyl *S*’-1-methylpropyl disulfide	0.08	5.34
**31**	C_7_H_14_S_2_	1177	1178	*S*-(*Z*)-1-propenyl *S*’-1-methylpropyl disulfide	2.28	39.00
**32**	C_7_H_14_S_2_	1182	1185	*S-*(*E*)-1-propenyl *S*’-1-methylpropyl disulfide	0.60	42.00
**33**	C_8_H_18_S_2_	1220	1225	Bis (1-Methylpropyl) disulfide	54.36	0.54
**34**	C_8_H_14_S_2_	1263	1265	Bis(1-methyl propenyl) disulfide	1.20	0.24
**35**	C_10_H_12_O	1281	1280	*t*-Anethole	0.00	0.42
**36**	C_8_H_18_S_4_	1426	1429	Bis(1-methyl thio)propyl disulfide	0.36	0.42
**37**	C_7_H_14_S_3_	1430	1435	*S*-(1-(methylthio)propyl) *S*’-(*Z*)-1-propenyl disulfide	0.05	4.50
**38**	C_7_H_14_S_3_	1433	1438	*S*-(1-(methylthio)propyl) *S*’-(*E*)-1-propenyl disulfide	0.00	0.48
**39**	C_15_H_24_	1452	1452	*α*-Humulene	0.00	0.05
**40**	C_15_H_24_	1560	1567	Germacrene B	0.06	0.00
				Sulfides	76.15	93.06
				Terpenoids	17.76	3.30
				Others	0.19	0.67
				Total identified	94.10	97.03

^a^ Retention index (RI) from the databases NIST and Wiley; ^b^ retention index determined with reference to a homologous series of *n*-alkanes on a ZB-5MS column. All metabolites were annotated at the MSI confidence level 2.

## Data Availability

Data will be made available on request.

## Supplementary Materials

The following supporting information can be downloaded at https://www.mdpi.com/article/10.3390/plants15182781/s1, Figure S1: Photographs of *F. foetida* and *F. tadshikorum* taken by *O. Khojimatov*; Figure S2: Oleo-gum resin from the roots of *F. foetida*; Figure S3: Oleo-gum resin from the roots of *F. tadshikorum*; Figure S4: Distribution of *F. foetida* and *F. tadshikorum* in the territory of Uzbekistan; Figure S5: GC chromatogram of the volatile compounds obtained from the oleo-gum resins of *F. tadshikorum*. The numbers indicate annotated metabolites (Table 1); Figure S6: GC chromatogram of the volatile compounds obtained from the oleo-gum resins of *F. foetida*. The numbers indicate annotated metabolites (Table 1); Figure S7: RP-UHPLC-QqTOF-MS total ion chromatogram of the methanolic extract obtained from the oleo-gum resin of *F. tadshikorum* acquired in the negative (A) and positive (B) ion mode as an MS1 full scan (TOF-scan) in the *m*/*z* range of 50-1250. The numbers indicate annotated metabolites (Table 2); Figure S8: RP-UHPLC-QqTOF-MS total ion chromatogram of the methanolic extract obtained from the oleo-gum resin of *F. foetida* acquired in the positive (C) and negative (D) ion mode as an MS1 full scan (TOF-scan) in the *m*/*z* range of 50-1250. The numbers indicate annotated metabolites (Table 3); Figure S9: Chemical structures of the major components of the oleo-gum resin of *F. tadshikorum*; Figure S10: Chemical structures of the major components of the oleo-gum resin of *F. foetida*; Figure S11: (+) ESI-MS/MS spectra of *m*/*z* 441.2272 that was annotated as 8-acetoxy-5-hydroxyumbelliprenin (57); Figure S12: (+) ESI-MS/MS spectra of *m*/*z* 399.2188 that was annotated as galbanic acid (58); Figure S13: (+) ESI-MS/MS spectra of *m*/*z* 255.0436 that was annotated as foetisulfide A (77); Figure S14: Comparative 1H NMR spectra in DMSO-d6 to show the difference in the NMR spectral patterns of oleo-gum resins of (a) *F. foetida* and (b) *F. tadshikorum*; Table S1: Selected 1H NMR signals suitable for the quantification by line fitting in OGRFF and OGRFT (picked from 600 MHz frequency spectra); Table S2: NMR signal assignment for 2-{[(2E)-3-(methylthio)-2-propen-1-yl]disulfanyl}butane (92) in DMSO-d6 (600/151 MHz). Custom atom numbering was used, not in accordance with IUPAC rules; Table S3: NMR signal assignment for 2-{[(2Z)-3-(methylthio)-2-propen-1-yl]disulfanyl}butane (93) in DMSO-d6 (600/151 MHz). Custom atom numbering was used, not in accordance with IUPAC rules; Table S4: NMR signal assignment for 8-acetoxy-5-hydroxyumbelliprenin (57) in DMSO-d6 (600/151 MHz). Custom atom numbering was used, not in accordance with IUPAC rules; Table S5: NMR signal assignment for galbanic acid (58) in DMSO-d6 (600/151 MHz). Custom atom numbering was used, not in accordance with IUPAC rules; Table S6: NMR signal assignment for coniferyl ferulate (45) in DMSO-d6 (600/151 MHz). Custom atom numbering was used, not in accordance with IUPAC rules.

## Abbreviations

The following abbreviations are used in this manuscript:

HS-GC-MS	Headspace gas chromatography–mass spectrometry
NMR	Nuclear magnetic resonance
GC-MS	Gas chromatography–mass spectrometry
RP-UHPLC-ESI-QqTOF-MS/MS	Reversed-phase ultra-high-performance liquid chromatography–electrospray ionization–quadrupole time-of-flight tandem mass spectrometry
GC-EI-Q-MS	Gas chromatography–electron ionization–quadrupole mass spectrometry
OGRFT	oleo-gum resins of *F. tadshikorum*
OGRFF	oleo-gum resins of *F. foetida*
