# Supplementary material for: Combination of GC-MS, UHPLC-MS, and NMR Reveals Metabolic Differences Between the Oleo-Gum Resins of Ferula tadshikorum Pimenov and F. foetida (Bunge) Regel
Source: Plants (Basel). 2026 Sep 11;15(18):2781. doi: 10.3390/plants15182781 (PMC13609968; doi:10.3390/plants15182781)
Supplement: Supplementary file 1 [file plants-15-02781-s001.zip › plants-4310553-supplementary.pdf]

Supplementary Information

Combination of GC-MS, UHPLC-MS, and NMR Reveals Metabolic Differences Between the Oleo-Gum Resins of Ferula tadshikorum Pimenov and F. foetida (Bunge) Regel

Nilufar Z. Mamadalieva [1,2,3*], Michal Šoral [4], Olim K. Khojimatov [5], Davlat K. Akramov [1], Andrej Frolov [3,6] and Ludger A. Wessjohann [3*]

1 Institute of the Chemistry of Plant Substances, Uzbekistan Academy of Sciences, M. Ulugbek Str 77, Tashkent 100170, Uzbekistan; n.mamadaliyeva@afu.uz (N.Z.M.); a.davlat@inbox.ru (D.K.A.)
2 Department of Pharmacy and Chemistry, Faculty of Dentistry and Pharmacy, Alfraganus University, Yuqori Qoraqamish Str. 2a, Tashkent 100190, Uzbekistan
3 Department of Bioorganic Chemistry, Leibniz Institute of Plant Biochemistry, Weinberg 3, 06120 Halle (Saale), Germany; afrolov@ipb-halle.de
4 Analytical Department, Institute of Chemistry, Slovak Academy of Sciences, Dúbravská cesta 9, SK-845 38 Bratislava, Slovakia; michal.soral@savba.sk
5 Institute of Botany, Uzbekistan Academy of Sciences, Durmon Yuli Str 32, Tashkent 100125, Uzbekistan; olimchik@mail.ru
6 Laboratory of Analytical Biochemistry and Biotechnology, K.A. Timiryazev Institute of Plant Physiology of Russian Academy of Science, Botanicheskaya street 35, 127276 Moscow, Russia

* Correspondence: wessjohann@ipb-halle.de

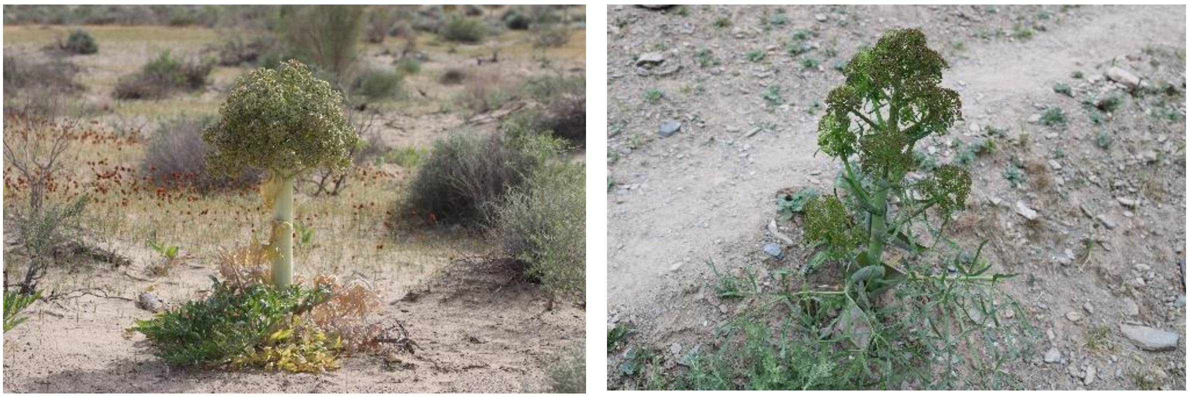

Ferula foetida *Ferula tadshikorum*

Figure S1. Photographs of *F. foetida* and *F. tadshikorum* taken by O. Khojimatov.

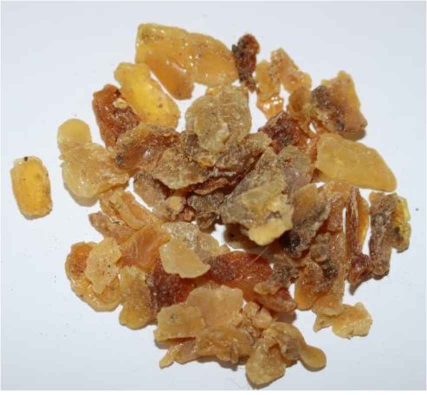

Figure S2. Oleo-gum resin from the roots of *F. foetida*

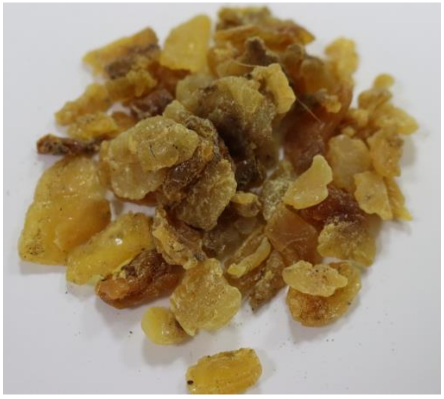

Figure S3. Oleo-gum resin from the roots of *F. tadshikorum*

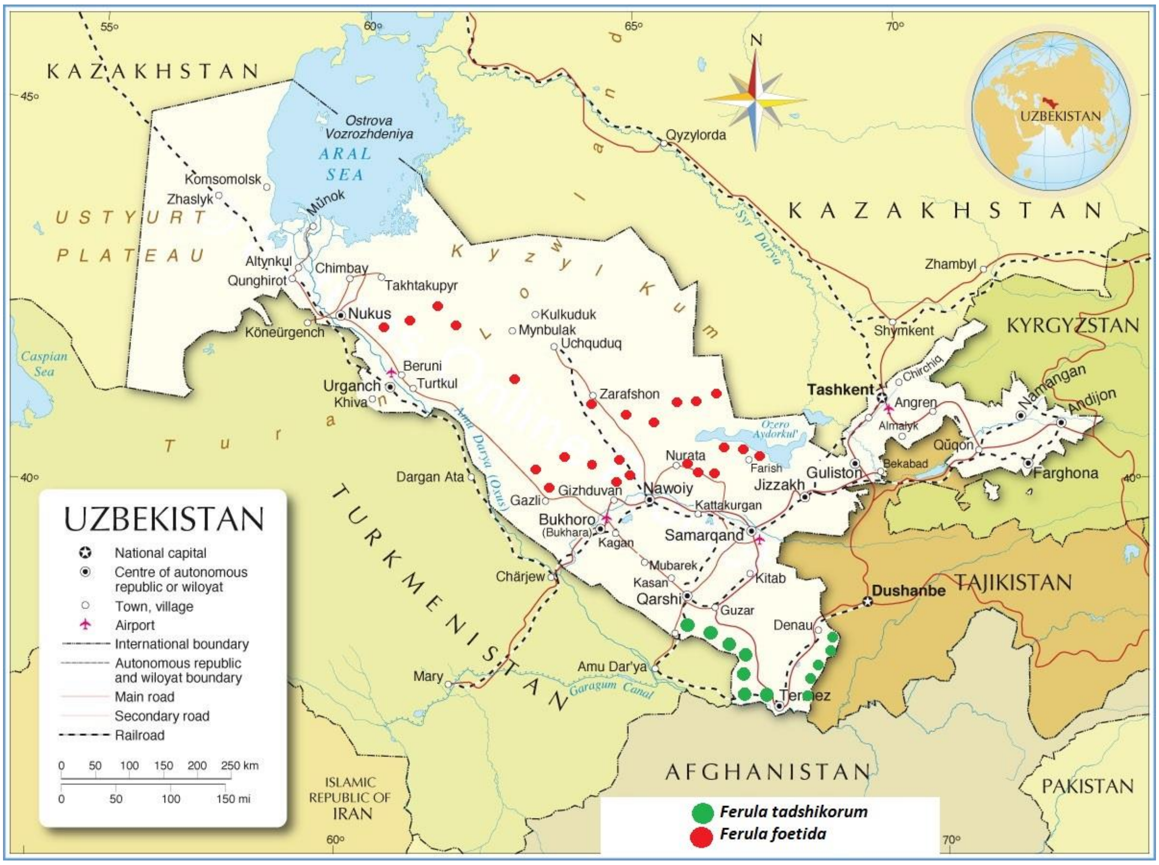

Figure S4. Distribution of *F. foetida* and *F. tadshikorum* in the territory of Uzbekistan

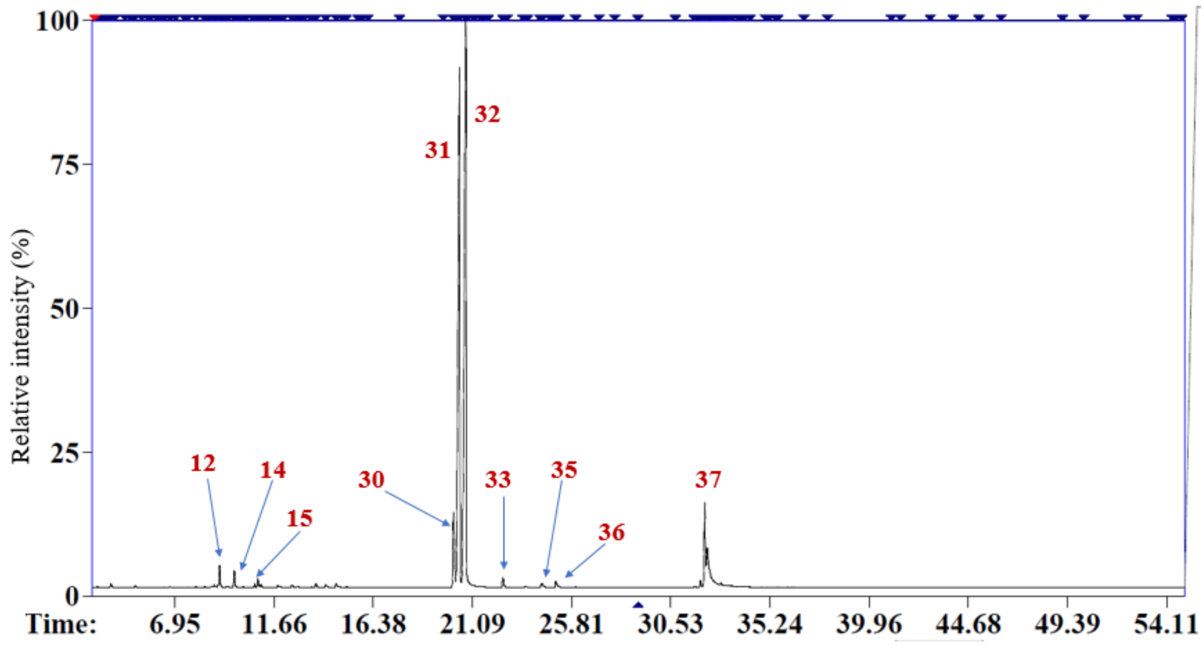

Figure S5. GC-chromatogram of the volatile compounds obtained from the of the oleo-gum resins of *F. tadshikorum.* The numbers indicate annotated metabolites (Table 1).

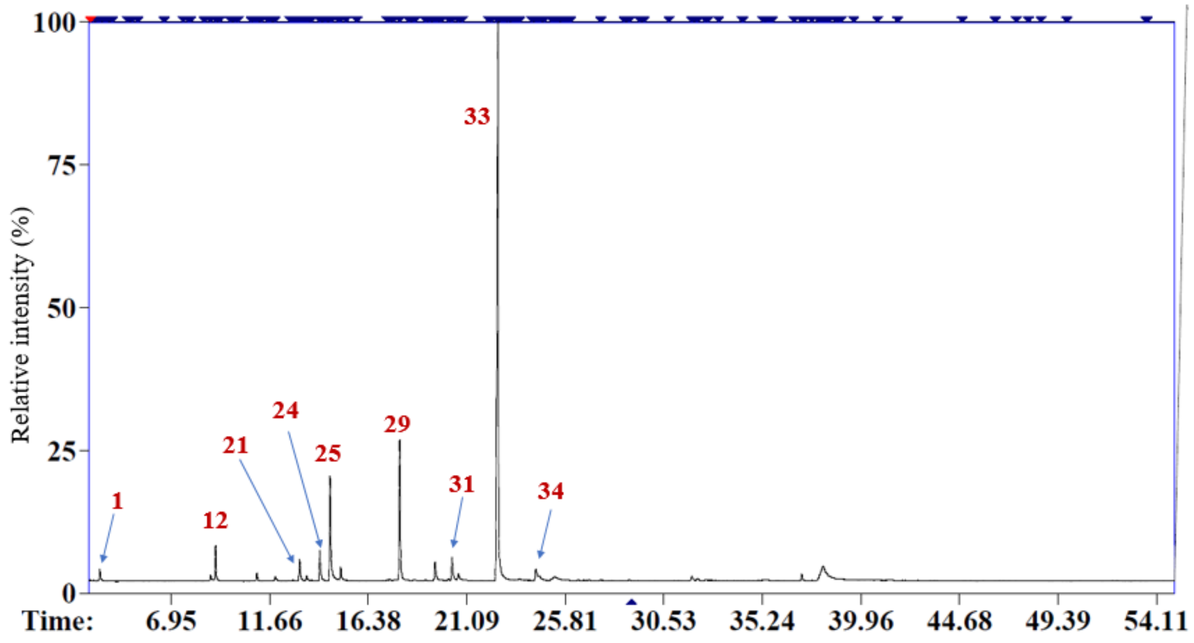

Figure S6. GC -chromatogram of the volatile compounds obtained from the of the oleo-gum resins of *F. foetida.* The numbers indicate annotated metabolites (Table 1).

A)

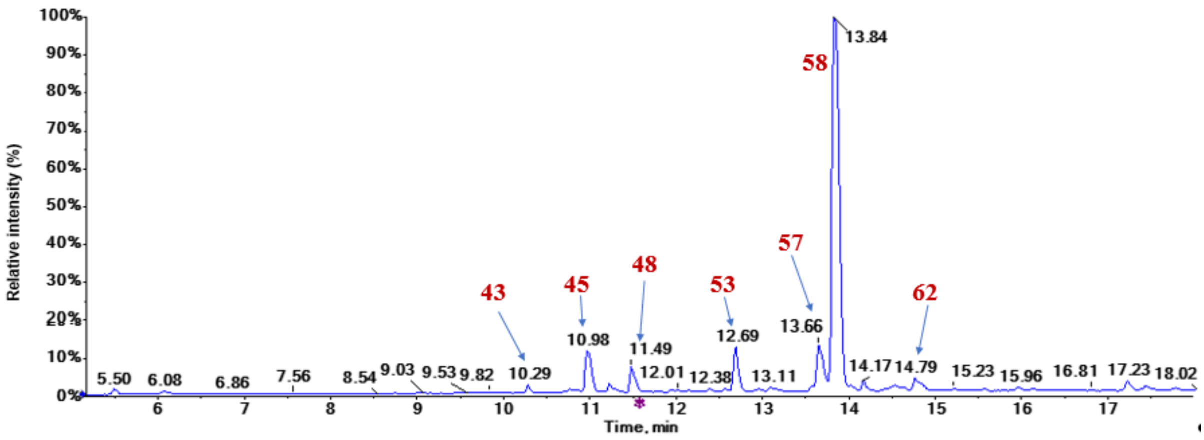

B)

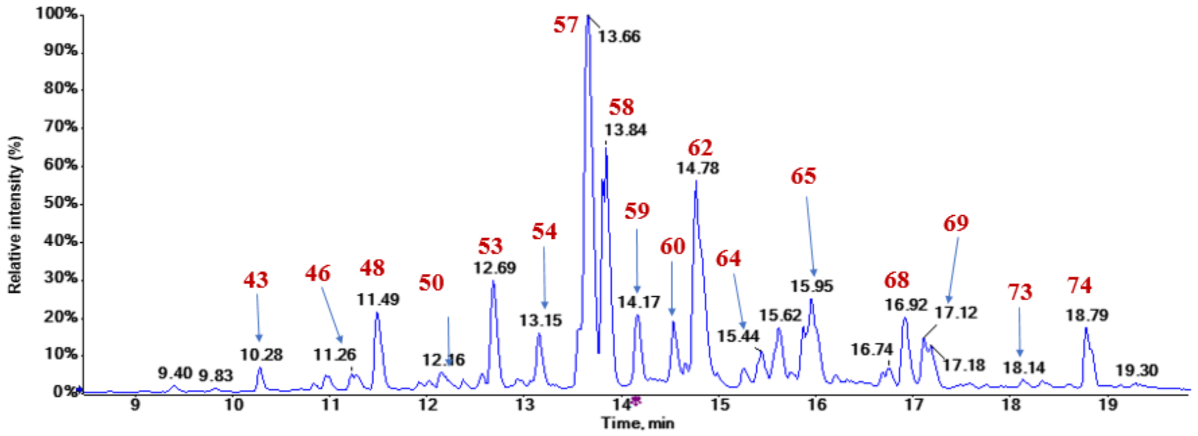

Figure S7. RP-UHPLC-QqTOF-MS total ion chromatogram of the methanolic extract obtained from the oleo-gum resin of *F. tadshikorum* acquired in the negative (A) and positive (B) ion mode as an MS1 full scan (TOF-scan) in the *m*/*z* range of 50-1250. The numbers indicate annotated metabolites (Table 2).

C)

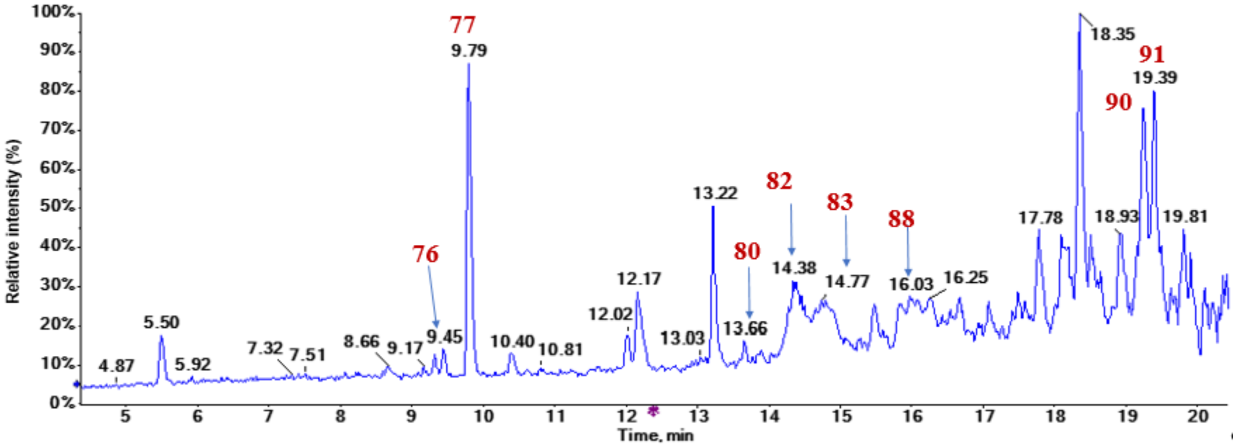

D)

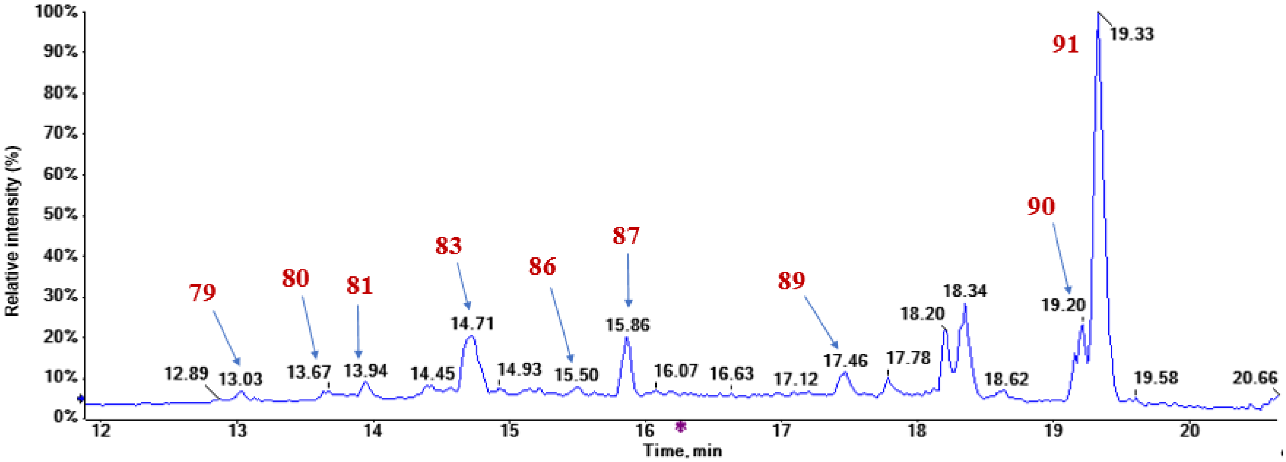

Figure S8. RP-UHPLC-QqTOF-MS total ion chromatogram of the methanolic extract obtained from the oleo-gum resin of *F. foetida* acquired in the positive (C) and negative (D) ion mode as an MS1 full scan (TOF-scan) in the *m/z* range of 50-1250. The numbers indicate annotated metabolites (Table 3).

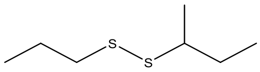

S-propyl,*S*′-1-methylpropyl disulfide (**30**)

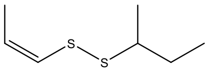

S-(*Z*)-1-propenyl,*S*′-1-methylpropyl disulfide (**31**)

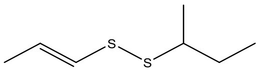

S-(*E*)-1-propenyl, *S*′-1-methylpropyl disulfide (**32**)

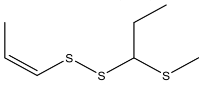

S-(1-(methylthio)propyl) *S*′-(*Z*)-1-propenyl disulfide (**37**)

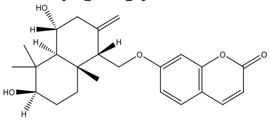

Assafoetidnol A (**48**)

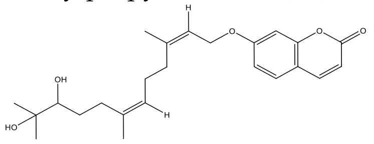

Karatavicinol (**53**)

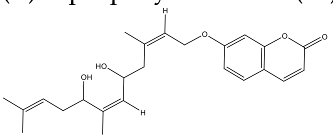

5,8-Dihydroxyumbelliprenin (**54**)

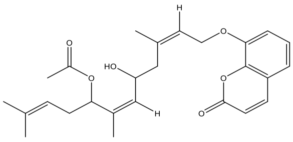

8-Acetoxy-5-hydroxyumbelliprenin (**57**)

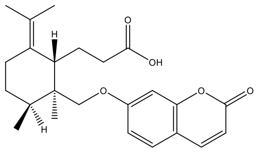

Galbanic acid (**58**)

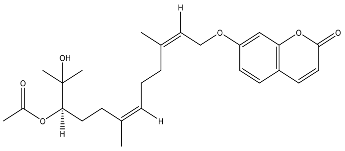

10'*R*-Acetoxy-11'-hydroxyumbelliprenin (**59**)

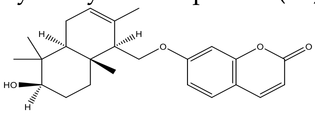

Feselol (**65**)

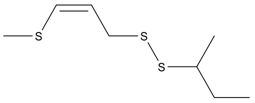

2-{[(2*Z*)-3-(methylthio)-2-propen-1-yl] disulfanyl}butane (**93**)

Figure S9. Chemical structures of the major components of the oleo-gum resin of *F. tadshikorum*

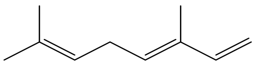

(*E*)-*β*-Ocimene (**25**)

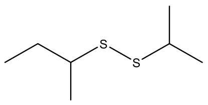

2-(Isopropyl disulfanyl)butane (**29**)

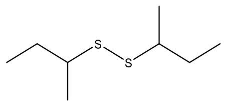

Bis (1-Methylpropyl) disulfide (**33**)

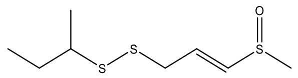

Foetisulfide A (**78**)

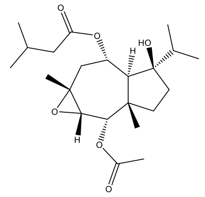

1-Acetate lapiferol 5-isovalerate (**83**)

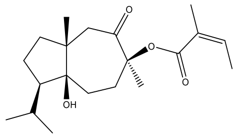

Felikiol 3-angelate (**87**)

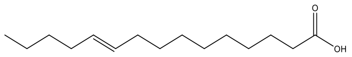

Pentadecenoic acid (**89**)

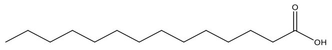

Palmitic acid (**90**)

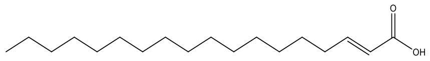

Octadecenoic acid (**91**)

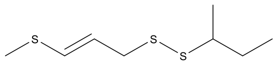

2-{[(2*E*)-3-(methylthio)-2-propen-1-yl] disulfanyl} butane (**92**)

Figure S10. Chemical structures of the major components of the oleo-gum resin of *F. foetida*

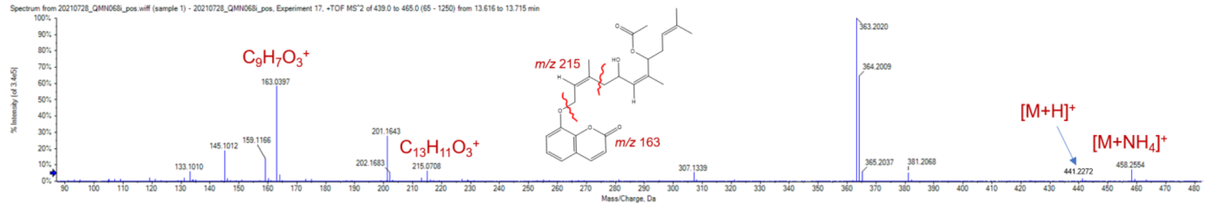

Figure S11. (+) ESI-MS/MS spectra of *m/z* 441.2272 that was annotated as 8-acetoxy-5-hydroxyumbelliprenin (**57**)

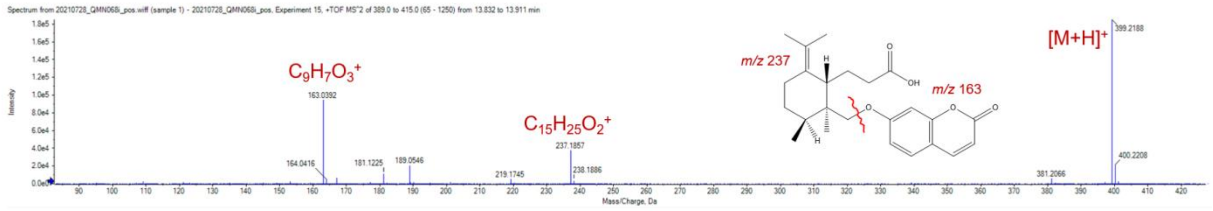

Figure S12. (+) ESI-MS/MS spectra of *m/z* 399.2188 that was annotated as galbanic acid (**58**)

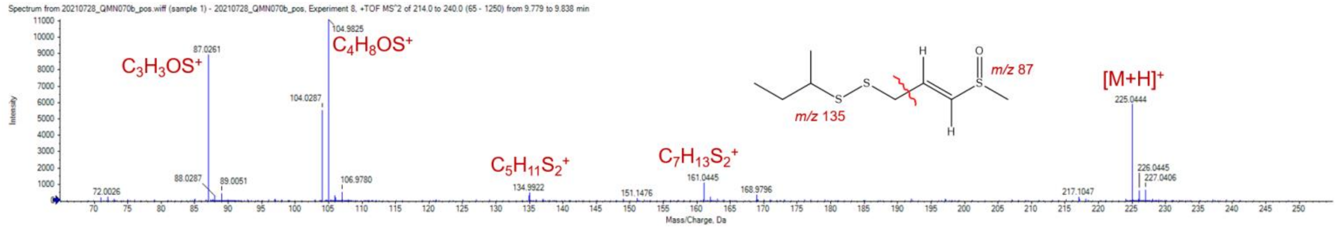

Figure S13. (+) ESI-MS/MS spectra of *m/z* 255.0436 that was annotated as foetisulfide A (**77**)

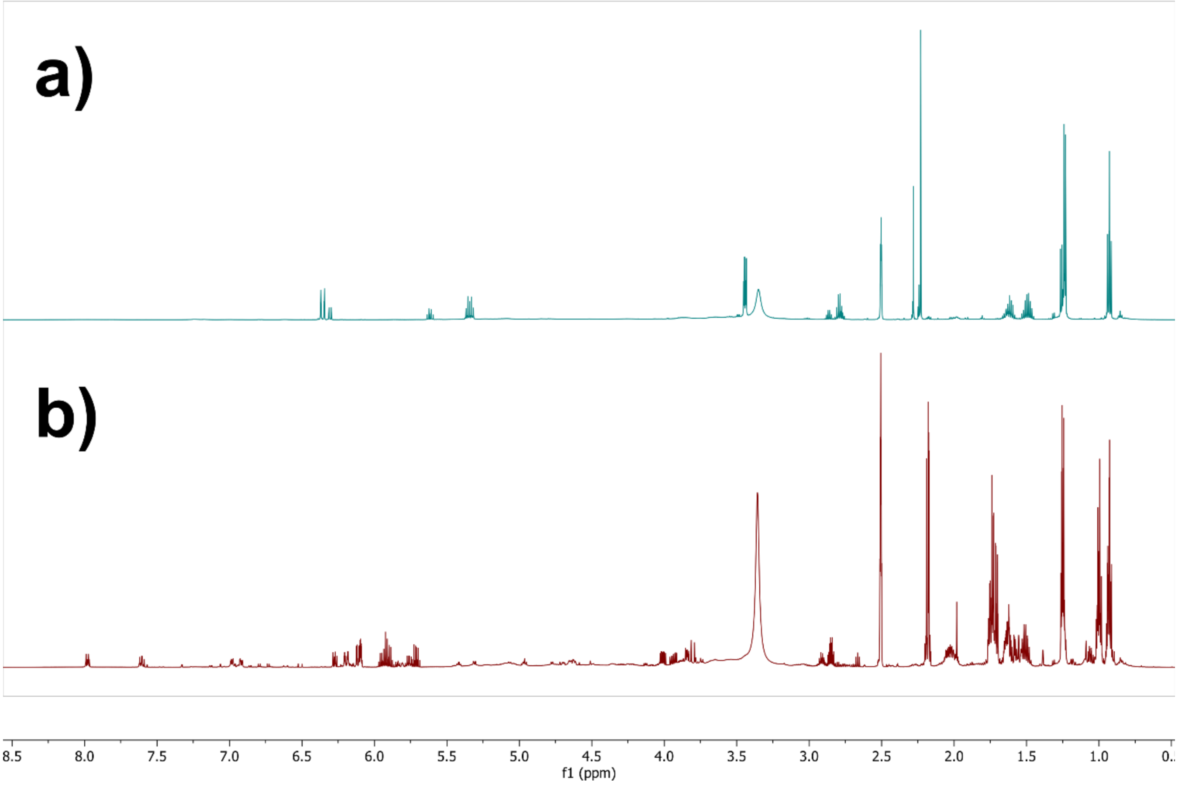

Figure S14. Comparative ^{1}H NMR spectra in DMSO-d_6 to show the difference in the NMR spectral patterns of oleo-gum resins of a) *F. foetida* and b) *F. tadshikorum*

Table S1. Selected ^{1}H NMR signals suitable for the quantification by line fitting in OGRFF and OGRFT (picked from 600 MHz frequency spectra)

**Oleo-gum resin**	**Compound**	**δ_H / ppm**
OGRFF	**92**	2.230*
		6.357
		5.342
	**93**	2.280*
		6.306
		5.615
		2.863
OGRFT	**45**	6.512
		6.858
		7.130
		7.329
	**57**	4.969†
	**58**	1.387*

Note: * CH_3-group; † Two overlapped CH-group signals.

Table S2. NMR signal assignment for 2-{[(2*E*)-3-(methylthio)-2-propen-1-yl]disulfanyl}butane (**92**) in DMSO-*d*6 (600/151 MHz). Custom atom numbering was used, not in accordance with IUPAC rules

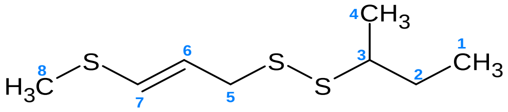

Position	δ_H / ppm	multiplicity, J_{HH} / Hz	δ_C / ppm
1	0.928	t, 7.4	11.2
2	1.492	m	28.3
	1.616	m	
3	2.793	sext, 6.8	47.0
4	1.235	d, 6.8	19.9
5	3.440	dd, 7.7, 1.1	41.4
6	5.342	dt, 14.8, 7.7	119.5
7	6.357	dt, 14.7, 1.1	129.1
8	2.230	s	13.7

Table S3. NMR signal assignment for 2-{[(2Z)-3-(methylthio)-2-propen-1-yl]disulfanyl}butane (**93**) in DMSO-d_6 (600/151 MHz). Custom atom numbering was used, not in accordance with IUPAC rules

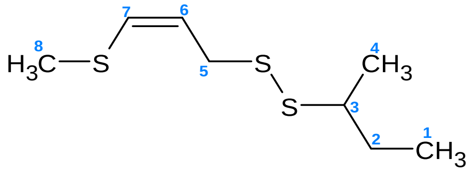

Position	δ_H / ppm	multiplicity, J_{HH} / Hz	δ_C / ppm
1	0.930	t, 7.4	11.2
2	1.509	m	28.2
	1.643	m	
3	2.863	sext, 6.8	46.9
4	1.260	d, 6.8	19.9
5	3.445	dd, 7.9, 0.9	37.4
6	5.615	dt, 9.3, 7.9	122.3
7	6.306	dt, 9.3, 0.9	132.2
8	2.280	s	16.2

Table S4. NMR signal assignment for 8-acetoxy-5-hydroxyumbelliprenin (**57**) in DMSO-d_6 (600/151 MHz). Custom atom numbering was used, not in accordance with IUPAC rules

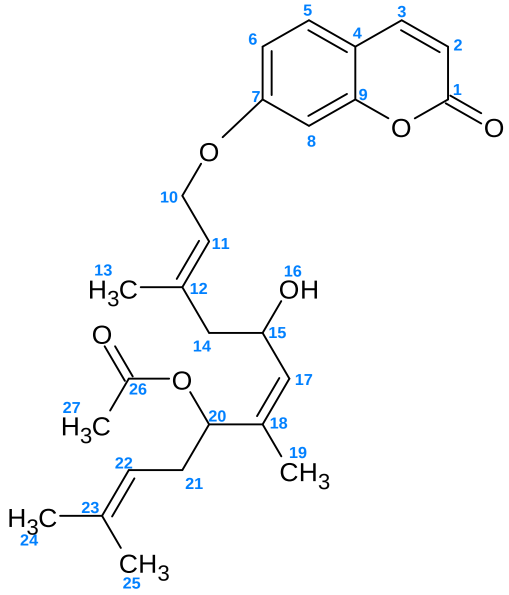

Position	δ_H / ppm	multiplicity, J_{HH} / Hz	δ_C / ppm
1	-	-	160.2
2	6.277	d, 9.5	112.3
3	7.981	d, 9.5	144.2
4	-	-	112.1

5	7.611	d, 8.6	129.3
6	6.920	dd, 8.6, 2.5	112.8
7	-	-	161.6
8	6.976	d, 2.5	101.2
9	-	-	155.3
10	4.633	m	65.1
11	5.417	tq, 6.6, 1.2	120.9
12	-	-	138.2
13	1.732	d, 1.2	17.0
14	2.041	m	47.3
	2.209	m	
15	4.360	m	65.1
16	4.699	d, 5.1	-
17	5.309	dq, 8.5, 1.2	131.9
18	-	-	132.4
19	1.585	d, 1.2	12.1
20	4.962	m	77.8
21	1.554	m	31.1
	1.626	m	
22	4.966	m	119.1
23	-	-	133.3
24	1.625	br s	25.4
25	1.554	br s	17.6
26	-	-	169.3
27	1.981	s	20.8

Table S5. NMR signal assignment for galbanic acid (**58**) in DMSO-d_6 (600/151 MHz). Custom atom numbering was used, not in accordance with IUPAC rules

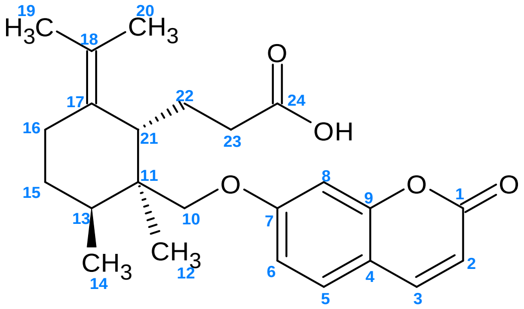

Position	δ_H / ppm	multiplicity, J_{HH} / Hz	δ_C / ppm
1	-	-	160.2
2	6.267	d, 9.5	112.2
3	7.975	d, 9.5	144.3
4	-	-	112.0
5	7.595	d, 8.6	129.2
6	6.917	dd, 8.6, 2.5	112.8
7	-	-	162.3
8	6.990	d, 2.5	100.9
9	-	-	155.4

10	3.741	d, 8.9	71.6
	3.860	d, 8.9	
11	-	-	40.2
12	1.089	s	22.3
13	1.830	m	34.3
14	0.902	d, 7.0	15.6
15	1.259	m	31.2
	1.522	m	
16	1.848	m	24.0
	2.437	br d, 13.3	
17	-	-	129.8*
18	-	-	124.5*
19	1.387*	d, 1.7	19.9*
20	1.579*	br s	20.0*
21	2.915	m	41.7
22	1.289	m	28.8
23	1.997	m	31.6
24	-	-	174.7

* the signal assignment for positions 17 and 18, and 19 and 20 can be interchanged

Table S6. NMR signal assignment for coniferyl ferulate (**45**) in DMSO-d_6 (600/151 MHz). Custom atom numbering was used, not in accordance with IUPAC rules

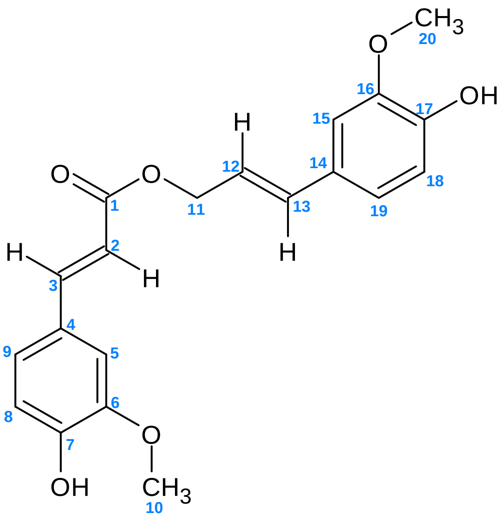

Position	δ_H / ppm	multiplicity, J_{HH} / Hz	δ_C / ppm
1	-	-	166.3
2	6.512	d, 15.9	114.3
3	7.582	d, 15.9	145.1
4	-	-	125.5
5	7.329	d, 2.0	111.2
6	-	-	147.8
7	-	-	149.2
8	6.794	d, 8.2	115.4
9	7.130	dd, 8.2, 2.0	123.0
10	3.814	s	55.6

11	4.774	dd, 6.5, 1.3	64.5
12	6.247	dt, 15.8, 6.5	120.4
13	6.613	dt, 15.8, 1.3	133.8
14	-	-	127.4
15	7.063	d, 2.0	109.5
16	-	-	147.6
17	-	-	146.7
18	6.732	d, 8.1	115.3
19	6.858	dd, 8.1, 2.0	120.0
20	3.791	s	55.5
